# Deciphering the nature and statistical optimization of antimicrobial metabolites of two endophytic bacilli

**DOI:** 10.1186/s13568-024-01811-3

**Published:** 2025-01-13

**Authors:** Raghda S. Isleem, Ahmed M. Eid, Saad El-Din Hassan, Khaled M. Aboshanab, Ghadir S. El-Housseiny

**Affiliations:** 1 Department of Microbiology and Immunology, Faculty of Pharmacy, Ain Shams University, Cairo, 11566 Egypt; 2 Department of Botany and Microbiology, Faculty of Science, Al-Azhar University, Cairo, 11884 Egypt

**Keywords:** Endophytes, Garlic, Bacillomycin, Mycosubtilin, Fengycin, Macrolactin H, And bacillibactin

## Abstract

In this study, *Allium sativum*, garlic, was selected to isolate endophytic bacteria and to evaluate the antimicrobial, antiviral, antioxidant, and cytotoxic activities of their produced metabolites followed by identification of the biosynthetic gene cluster of the antimicrobial metabolites using Oxford Nanopore Technology (ONT). Two bacterial isolates, C6 and C11, were found to have a broad-spectrum antagonistic effect against four standard microbial strains and were molecularly identified using 16 S ribosomal RNA sequence analysis and deposited in a local culture collection as *B. velezensis* CCASU-C6, and *B. subtilis* CCASU-C11, respectively. Optimization for the maximum production of antimicrobial metabolites revealed that a four-day incubation period was optimal, with sucrose and tryptone serving as the best carbon and nitrogen sources for the fermentation media. Response surface methodology model using the central composite design was created resulting in a 1.2-fold and 1.8-fold improvement in antimicrobial metabolite(s) production of C6 and C11 isolates, respectively. The optimal production conditions were found to be a temperature of 33 °C, pH of 7, and an agitation rate of 200 rpm for C6 metabolite, and a temperature of 37 °C, pH of 7, and an agitation rate of 250 rpm for C11 metabolite. Both bacterial isolates displayed antioxidant and antiviral activity and mild cytotoxic action. Genomic sequence and antiSMASH analysis showed that the biosynthetic gene clusters of bacillomycin, mycosubtilin, fengycin, and macrolactin H in *B. velezensis* CCASU-C6 and bacillibactin and Macrolactin H in *B. subtilis* CCASU-C11 showed 100% conservation.

**Supplementary Information:**

The online version contains supplementary material available at 10.1186/s13568-024-01811-3.

## Introduction

The development of antibiotic resistance by pathogenic bacteria and fungi, the insufficient efficacy of current antibacterial and antifungal agents for various bacterial and fungal infections, and the evolution of life-threatening viruses necessitate an urgent search for new sources of novel and effective antimicrobial agents (Monowar et al. 2018). Antibiotic resistance is created by several factors, including incorrect and unnecessary antibiotic use, inadequate sanitation, delayed infection diagnosis, and immunocompromised patients (Subramani et al. 2017). Medicinal plants are the predominant sources of biologically active substances utilized in current medication production and development (Newman 2018). Over 80% of medications are currently derived from medicinal plants (Nxumalo et al. 2020). Medicinal plants refer to whole plants or parts containing active ingredients or biologically active secondary metabolites. Herbal medicines are pharmaceutical preparations that contain active metabolites extracted from herbs and plants (Abubakar and Haque 2020). The World Health Organization (WHO) believes that 80% of the world’s human population relies on traditional medicines derived from plants for remedies for diseases, including gastrointestinal diseases, urinary tract infections, asthma, respiratory tract disorders, skin diseases, and cardiovascular diseases (Egamberdieva et al. 2017). Herbal medicine has many advantages as natural products are considered inexpensive, safer, less toxic, and easily accessible (Zafar et al. 2019; Abubakar and Haque 2020). Although medicinal plants produce metabolites with strong physiological activities, their continuous production is facing troubles and difficulties due to stressful environmental circumstances and rapid climate fluctuations, which lead to reduced biodiversity in the medicinal plants. Thus, the discovery of bioactive metabolites from microorganisms (bacteria and fungi) dwelling in medicinal plants has become a promising substitute route for drug synthesis (Buatong et al. 2011). According to the gene transfer process between the host plants and their endophytes, the endophytes accept the same genetic information as their host plants and create secondary bioactive compounds with the same properties (Kusari et al. 2012). So, recently, research on discovering new curative agent resources has focused on endophytic microorganisms’ isolation from plants due to their multiple novel and interesting bioactive compounds (Sharma and Mallubhotla 2022).

The term endophyte expresses all non-pathogenic microorganisms (bacteria, fungi, and actinomycetes) that inhabit the interior of both below- and above-ground tissues (Afzal et al. 2019) of healthy plant tissues without inflicting adverse pathogenic consequences on the host plant (Midhun and Jyothis 2021). Almost all plants are assumed to be colonized by endophytic microbes. Endophytes are known to create useful symbiotic interactions with their hosts (Midhun and Jyothis 2021). The importance of endophytic bacteria has been demonstrated as sources of novel bioactive compounds for drug synthesis and development and is now widely recognized (Gupta et al. 2017). They exhibit various biological actions, including antibacterial, antioxidant, anti-inflammatory, antifungal, antiviral, and anticancer effects. Endophytes also create secondary metabolites that have diverse positive impacts on the host plants (Mushtaq et al. 2023; Stelmasiewicz et al. 2023).

Garlic (Allium Sativum L.), which belongs to the Alliaceae family, has been widely consumed as a valuable spice, vegetable, and a common effective medication for healing infectious disorders (Huang 2019). Traditionally, garlic was used due to its preventive mechanisms as antibacterial, antiviral, antifungal, antitumor, analgesic, antipyretic, and antioxidant features (El-Gayar et al. 2016; Wang et al. 2019; Nassar et al. 2023). Because of its numerous medicinal applications, garlic plant (*Allium sativum*) was chosen in this study to investigate its associated endophytic bacteria. The isolated bacteria were screened for secondary metabolites with antibacterial and antifungal activity. Moreover, its biological activities were also evaluated.

## Materials and methods

### Collection of the plant sample

Fresh and mature garlic roots, leaves, and cloves were carefully cut out and harvested from agricultural land in El-Menofia governorate, Egypt (30° 38′ 40.9″ N 30° 56′ 49.9″ E) in April 2021. The samples were collected in sterilized polythene zip-lock bags and promptly delivered to the botany department’s laboratory at Al-Azhar University in Cairo, where they were preserved at 4 °C in a refrigerator till usage within 24 h after collection.

### Isolation of endophytic bacteria

#### Surface sterilization

The collected garlic (roots, leaves, and cloves) was cleaned thoroughly under running tap water to eliminate dust, and soil remains from the plant surface, and air dried. Then, the plant parts were sterilized by being submerged for 1 min in sterile distilled water, then dipped in ethanol 70% for 1 min, followed by being washed in 2.5% sodium hypochlorite for 4 min, ethanol 70% for 30 s, and lastly rinsed three times in sterile distilled water. To evaluate the effectiveness of the plant surface sterilization, distilled water from the final washing was spread onto a nutrient agar plate, followed by incubation for 24 h at 37 ºC. The efficacy of the plant surface sterilizing procedure has been verified by the lack of microbial colonies on the incubated plates (Ismail et al. 2021; Stelmasiewicz et al. 2023).

#### Media for isolation of endophytic bacteria

Nutrient agar was utilized to isolate endophytic bacteria. Since the nutrient agar medium cannot suppress the endophytic fungi growth, it was treated with nystatin (30 µg/mL) to suppress the fungal growth.

#### Isolation and purification of endophytic bacteria

After proper drying of surface sterilized garlic parts (leaves, roots, and cloves), a sterile scalpel was used to cut some of these parts into small pieces 5 mm long in a laminar airflow cabinet and each piece was spread on a surface of the prepared nutrient agar plate. Other parts were ground in a sterile saline solution using a sterile pestle and mortar in the laminar airflow cabinet. About 1 mL of the ground sample was diluted, and a sterile glass -rod was used to spread 0.1 mL of 10^− 2^ diluted solution onto the nutrient agar plates. All plates were then incubated for 24–72 h at a temperature of 37 ºC (Ismail et al. 2021). After 72 h, bacterial growth was observed on the plates. To obtain pure bacterial isolates, a sterile inoculation loop was used to collect a single colony from each plate and streaked onto fresh nutrient agar plates then incubated at 37 ºC. Then all different isolates were sub-cultured into nutrient broth with 50% glycerol and then kept in a freezer at -80 °C till further experimental use (Sharma and Mallubhotla 2022).

### Preliminary identification of endophytic bacterial isolates

All endophytic bacterial isolates were identified through morphological standards, including colony shape and color, as well as microscopic characterization like Gram staining and biochemical tests.

### Production of bioactive metabolites

Pure bacterial endophyte isolates were pre-cultured on nutrient agar at 37 °C overnight. To prepare the seed culture, a single colony of each bacterial isolate was cultured in 30 mL of sterilized nutrient broth and incubated overnight at 33 ºC on a shaker at 150 rpm. Subsequently, 0.5 ml of the seed culture was utilized to inoculate 50 ml of nutrient broth, which was incubated for 7 days at 33 °C and 150 rpm. After 7 days, all culture broths were centrifuged for 20 min at 8000 rpm, and then the supernatant of culture broth was used to screen the antibacterial and antifungal activity for each isolate (Balouiri et al. 2016; Ebu et al. 2023).

### Evaluation of the antibacterial and antifungal activity

All endophytic isolates were evaluated for antibacterial activity using Muller-Hinton agar medium (MHA) through the agar well diffusion technique as previously described (Balouiri et al. 2016). The bacterial test was performed against the following standard strains, *B. subtilis* ATCC 6633 and *S. aureus* ATCC 6538, *E. coli* ATCC 8739, and *P. aeruginosa* ATCC 9022. The antifungal activity was evaluated against *C. albicans* ATCC10231. A fresh culture of the test organisms was adjusted to a count of 0.5 McFarland and was spread by a sterile cotton swab on the surface of the prepared MHA. Then, wells (6 mm) were made in MHA plates using a sterile cork-borer, and 150 µL of supernatant obtained from centrifuged endophyte broth was pipetted to each well individually. The plates were left at room temperature for 15 min to allow diffusion of the supernatant in the agar. The plates were then incubated at 37 °C for 18 h. After incubation, the diameter of the inhibition zone surrounding the wells was measured in mm. The experiment was carried out in triplicate (Sharma and Mallubhotla 2022; Nassar et al. 2023).

### Molecular identification of the selected isolates

The selected endophytic isolates were identified using 16 S ribosomal RNA sequence analysis (Clarridge 2004). The PCR products were sequenced at the GATC Biotech (A company using a DNA sequencer (ABI-3730xl) as a partner of Sigma Aldrich, Cairo, Egypt). The resulting sequences were compared with that available in the GenBank with the Basic Local Alignment Search Tool (https://blast.ncbi.nlm.nih.gov/Blast.cgi) to determine the closest related species. The final sequence was aligned using the ClustalX 1.8 software package (http://wwwigbmc.u-strasbg.fr/BioInfo/clustalx). A phylogenetic tree was organized using MEGA (Version 6.1) software based on the neighbor-joining method (Kumar et al. 2018). The confidence level of branching and clustering patterns (1,000 replications) was assessed by bootstrap analysis.

### **Factors affecting the production of biologically active metabolites**

The effects of the incubation time, fermentation medium composition, and environmental parameters such as incubation temperature, media pH, and agitation rate were investigated for maximum production of the bioactive metabolite(s).

#### Incubation time

A time course experiment was used to evaluate the optimal time for producing antimicrobial metabolites with the greatest growth inhibition against the tested strains. For each isolate, 10 Erlenmeyer flasks (250 mL) having 50 mL of nutrient broth were used. Then, 0.5 ml of the seed culture prepared above was inoculated into the flasks and cultured at 33 ºC for 10 days with 150 rpm agitation and a pH of 7. Each day, one flask was withdrawn from the incubator, centrifuged, and about 150 µL of the supernatant was obtained for evaluating the antimicrobial activity as mentioned above.

#### Carbon sources

For each bacterial isolate, three Erlenmeyer flasks (250 mL) with 50 mL of the nutrient broth containing two grams of either sucrose, galactose, or starch were used. Then, the respective culture broth was inoculated with 0.5 mL of the seed culture prepared as mentioned above. The flasks were then incubated at 33 ºC for 4 days with an agitation rate of 150 rpm with adjusted initial pH of 7. After incubation, the obtained broths were centrifuged, and 150 µL was used for evaluating the antimicrobial activity as mentioned above.

#### Nitrogen sources

For each bacterial isolate, sodium nitrate, tryptone, and beef extract at a concentration of 2 g/L were added individually to three Erlenmeyer flasks (250 mL) with 50 mL of the nutrient broth containing the carbon source that gave maximum production of the bioactive metabolites. Each flask was inoculated with 0.5 mL of the seed culture prepared as mentioned before and incubated at 33 ºC for 4 days at 150 rpm and adjusted initial pH of 7. After incubation, the obtained broths were centrifuged, and 150 µL was used to evaluate the antimicrobial activity as mentioned above.

#### Optimizing environmental factors

The effect of different environmental factors such as temperature (A), pH (B), and agitation rate (C) on metabolite production was optimized using a central composite design (CDD) from response surface methodology. In total, 16 runs were performed for C6 isolate against *B. subtilis* and 16 runs for C11 isolate against *C. albicans*, using the optimal carbon and nitrogen sources, and the factors and levels used for these experiments are as follows: temperature: 29, 33, and 37 ºC, pH: 6, 7 and 8 and agitation rate: 150, 200 and 250 rpm. A single response, IZ diameter (mm), was determined after incubation for 4 days. The mean of three measurements was recorded for each run. The design of experiments was carried out by Design Expert^®^ v. 7.0 (Design Expert^®^ Software, Stat-Ease Inc., Statistics Made Easy, Minneapolis, MN, USA). To validate the obtained model, ANOVA (Analysis of variance) was used. Optimum conditions were obtained using the numerical optimization function in the same software (Thompson et al. 1999).

#### Diagnostic plots

Four plots were built for each isolate to validate our models. A normal probability plot reveals if the residuals stick to a normal distribution. Box-Cox plot on the other hand is used to decide the best power transformation. The predicted vs. actual plot shows how well the model predicts our responses. Finally, Residuals vs. Run plot tests for lurking variables that may have altered the results during the experiments (Design Expert Version 7 User’s Guide).

### Evaluation of the antiviral, antioxidant, and cytotoxic activity

#### Extraction of the bioactive metabolites

About 3 mL of the seed culture of the selected isolate was inoculated in 300 mL of nutrient broth containing the optimal carbon, nitrogen sources, and environmentally optimized as described above. The obtained broth cultures were centrifuged at 8000 rpm for 20 min, and the liquid supernatant was mixed with the same volume of ethyl acetate and shaken strongly for 30 min. The mixture was filled in a separating funnel to separate the phase of the organic solvent containing bioactive metabolites. Then, the organic solvent was evaporated by a rotary evaporator at 50 ºC to yield the crude extract. For antiviral, antioxidant, and cytotoxic assays, 1 mL of dimethyl sulfoxide (DMSO) was utilized to dissolve the crude extract (Mamarasulov et al. 2023).

#### Antioxidant assay

The antioxidant effectiveness of the crude extract of metabolites produced by endophytic bacteria was assessed using the 1, 1- diphenyl-2-picryl hydrazyl (DPPH) free radical scavenging technique. During this procedure, nine concentrations of each sample were prepared as follows, 1000, 500, 250, 125, 62.5, 31.25, 15.62, 7.81, and 3.9 µg/mL. Then about 3 mL of the prepared solution was transferred to a test tube containing one mL of DPPH solution (0.1 mM) and rapidly shaken before being incubated for 30 min at room temperature. Under the same conditions, ascorbic acid was used as a positive control. A spectrophotometer was used to measure the absorbance of the DPPH radical solutions at 517 nm. The DPPH free radical scavenging percent was calculated by the following equation:

$$ {\text{DPPH}}\;{\text{scavenging}}\;{\text{activity}}\;\% = \left[ {{\text{Ao}}{-}{\text{ A1}}/{\text{Ao}}} \right] \times {1}00 $$

Ao = absorbance of the control at 517 nm, and A1 = the sample absorbance. The extract’s median scavenging concentration (IC50) of the extract against DPPH was calculated graphically as previously reported (Sulistiyani et al. 2016).

#### Assay of cytotoxicity

The cytotoxicity of the crude extract of metabolites produced by the respective endophytic isolate was evaluated using the 3-(4, 5-dimethylthiazol-2-yl)-2, 5-diphenyl tetrazolium bromide) (MTT) cell proliferation assay against PC3 (prostatic small cell carcinoma) and Vero cells (Cercopithecus aeithiops kidney epithelial normal cell) according to William and Surin (2014).

In this assay, PC3 and Vero cells were individually inoculated into 96-well tissue culture plates with an intensity of 1 × 10^5^/ mL (100 µL / well) and incubated for 24 h at 37 °C. When the monolayer sheet had fully formed, it was rinsed two times with washing media. Different concentrations of the tested bacterial extracts were prepared by two-fold dilution (1000, 500, 250, 125, 62.5, and 31.25 µg/mL) in RPMI medium with 2% serum (as a maintenance medium). Following that, 0.1 ml of each prepared dilution was added to each well except for three wells (utilized as a control) and received 0.1 mL of the maintenance medium. The plate was then incubated at 37 °C for 24 h. After that, an inverted microscope was used to observe any physical indicators of toxicity such as cell granulation, shrinkage or rounding of the cell, or partial or total loss of monolayer cells. Then, 20 µL of MTT solution (5 mg/mL in phosphate-buffered saline solution, PBS) was added to the wells and shaken thoroughly for 5 minutes at 150 rpm before being incubated at 37 °C with 5% CO2 for four hours. After discarding the MTT solution, each well was filled with 200 µL of DMSO and shaken rapidly for 5 minutes at 150 rpm to suspend the formazan crystal (William and Surin 2014). ELISA reader 76 (Laboratory of Science Way for Scientific Researches and Consultations, AL-Moqattam, Cairo) was used to measure well absorbance (A) at 560 nm. The results were provided as cell viability percentages against untreated control cells. Triplicate wells were assayed, and standard deviations were calculated.

The cell viability and inhibition were measured as shown in Eqs. 1 and 2.

1 $$ {\text{cell}}\;{\text{viability}}\;\left( \% \right) = \left( {{\text{mean}}\;{\text{OD}}\;{\text{of}}\;{\text{test}}/{\text{mean}}\;{\text{OD}}\;{\text{of}}\;{\text{the}}\;{\text{control}}} \right) \times {1}00 $$

2 $$ {\text{inhibition }}\left( {{1}00\% } \right) \, = { 1}00 \, {-}{\text{ viability}}\% $$

#### Antiviral activity assay

The antiviral activity experiment was performed against *Herpes Simplex* virus type 1 (HSV1). Initially, the maximum non-toxic concentration (MNTC) of the tested extracts on Vero cells was evaluated using the MTT assay. After that, cells with an intensity of 1 × 10^4^ cells/200 µL were introduced to each well in a 96-well plate, except for three wells that were kept for blank controls. The plate was then incubated at 37 °C with 5% CO_2_ overnight, to allow adherence of cells to the wells. A suspension from an equal volume (1:1 v/v) of the virus and nonlethal dilution of the tested extract was prepared and incubated for one hour. Following that, the wells were filled with 100 µL of viral/sample suspension, agitated at 150 rpm for 5 min, then incubated for 24 h at a temperature of 37 °C with 5% CO_2_. Cell viability was calculated using the MTT method as explained above.

### Genomic analysis for identification of the metabolite biosynthetic gene clusters

#### DNA extraction

The Qiagen DNeasy power- kit (Qiagen, Hilden, Germany) was used to extract DNA in accordance with the manufacturer’s instructions. The Qubit fluorometer ver. 4.0 (Thermo Fisher Scientific, Waltham, Massachusetts, USA) was used to measure the concentration of DNA to confirm that it was at least 55 ng/µL, in accordance with the Oxford Nanopore Standard Operating Procedure (Eltokhy et al. 2021).

#### Library construction

A total of 34 µL sequencing buffer, 25.5 µL of loading beads, and 4.5 µL nuclease-free water were added and mixed with 12 µL DNA. A Rapid Sequencing Kit (ONT, Oxford, UK) was used to construct the library. Following the fabrication of the library, priming and loading into the FLO-MIN106 (Nanopore Technology, Oxford, England) flow cell was carried out (Eltokhy et al. 2021).

#### Sequencing and data analysis

The DNA Sequences were performed using the MinION™ (ONT, Oxford, UK). After a full day, 3.03 M reads are produced, and 9.29 K is the N50. Guppy generated real-time base calling during the sequencing process. FAST5 and FASTq files were the format of the output; readings lower than Q7 were not included. Centrifuge software was used to classify the sequences to taxonomy identities (Kim et al. 2016). The Centrifuge index was created using human reference genome (GRCh38), bacterial, and viral genomes that were retrieved from the National Centre of Biotechnology Information (NCBI) RefSeq. Low-complexity areas in the reference sequences with a dust score greater than 20 were masked using the NCBI’s Dust Masker (v1.0.0). The results were visualized using Re-centrifuge (Martí 2019).

#### Identification of the secondary metabolite gene cluster(s)

The Antibiotics and Secondary Metabolite Analysis Shell (AntiSMASH version 2) was used to align and analyze the sequences for likely secondary metabolite gene clusters (https://antismash.secondarymetabolites.org/#!/start (accessed on 10 June 2024). As previously mentioned, the genome comparison was drafted using the Mauve software (https://gel.ahabs.wisc.edu/mauve) (Eltokhy et al. 2024).

### Statistical analysis

All trials were carried out thrice; the error bars indicate the standard deviation. The obtained results were entered and analyzed in the Microsoft Excel program. The SAS version 9.1 software package was used for Analysis of variance (ANOVA) (Thompson et al. 1999). Statistically significant differences were demonstrated by *p* < 0.05. Design Expert v.7.0 was used for creating the experiment design, model diagnostic plots, response surfaces, and ANOVA analysis.

## Results

### Isolation and preliminary identification of endophytic isolates

A total of nineteen endophytic isolates were isolated as pure colonies from cloves (13 isolates), roots (3 isolates), and leaves (3 isolates) (Table 1). The recovered isolates were of a wide range of colony shapes, including round or irregular, were colored off-white, creamy, and white and all were catalase positive. Based on cell shape and Gram staining, the 19 isolates were recognized as Gram-positive bacilli.

**Table 1:** Antibacterial and antifungal activity of the recovered endophytic isolates

Isolate code	Mean Inhibition zones (IZ in mm) ± SD				
	E. coli ATCC 8739	*P*. aeruginosa ATCC 9022	B. subtilis ATCC 6633	S. aureus ATCC 6538	Candida albicans ATCC10231
C1	0	0	0	0	0
C2	0	0	0	13.0 ± 0.33	0
C3	0	0	19.0 ± 0.67	0	13.5 ± 0.3
C4	0	13.0 ± 0.33	0	0	0
C5	0	0	17.5 ± 0.33	16 ± 0.66	0
C6	0	12.0 **±** 0.33	15.0 ± 0.33	14.5 ± 0.33	12.0 ± 0.67
C7	0	0	0	0	0
C8	0	0	0	0	0
C9	0	0	0	0	0
C10	0	0	0	0	0
C11	0	16 ± 0.67	13.0 ± 0.67	13.0 ± 0.33	10.0 ± 0.33
C12	0	0	0	0	14.0 ± 0.33
C13	0	0	12.5 ± 0.67	0	0
R1	0	0	18.0 ± 0.33	15.5 ± 0.67	0
R2	0	0	0	13.0 ± 0.33	15.5 ± 0.25
R3	0	0	0	0	0
L1	0	0	15.0 ± 0.33	0	0
L2	0	0	15.0 ± 0.33	14.5 ± 0.33	0
L3	0	0	14.0 ± 0.67	0	0

* Cloves are denoted by C, roots by R, and leaves by L, SD, standard deviation

## Antimicrobial evaluation test

The average inhibition zone (IZ) in mm of each isolate is displayed in Table 1. According to the documented data in Table 1, all bacterial isolates did not exhibit any potential activity against *E. coli*. C4, C6, and C11 isolates displayed antibacterial activity against *P. aeruginosa*, whereas C3, C5, C6, C11, C13, L1, L2, and L3 had antibacterial activity against *B. subtilis*. C2, C5, C6, C11, R1, R2, and L2 showed antibacterial activity against *S. aureus*. The isolates C3, C6, C11, C12, and R2 showed antifungal activity against *C. albicans*. Antibacterial activity using the cup plate technique of the crude extract of the endophytic isolates C6 and C11 against (a) *P. aeruginosa* ATCC 9022, (b) *B. subtilis* ATCC 6633, (c) *S. aureus* ATCC 6538, (d) *C. albicans* ATCC10231 standard strains is depicted in Fig. S1. Therefore, the endophytic isolates C6 and C11 were selected for further experimental studies.

### Molecular identification and culture deposition of the bacterial isolates

**Fig. 1:**
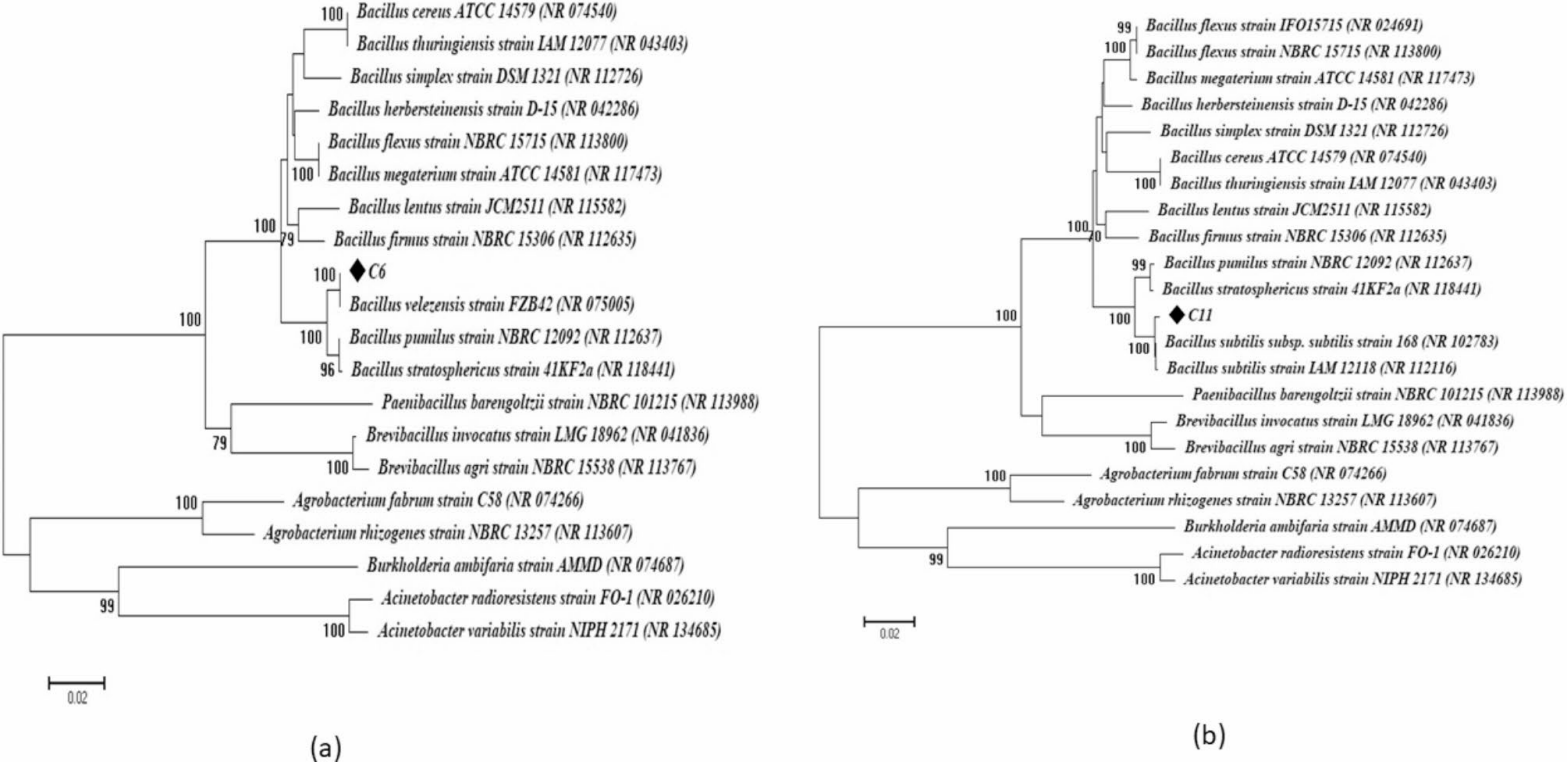
Phylogenetic tree based on 16 S ribosomal RNA gene sequence of** a** C6 and ** b** C11 endophytic bacteria isolated from the garlic clove

Based on the 16 S ribosomal RNA and phylogenetic analysis of the obtained DNA sequences of the C6 and C11 isolates, they were identified as *Bacillus velezensis* strain C6, and *Bacillus subtilis* strain C11, respectively (Fig. 1a &b). The respective nucleotide sequences were submitted into the NCBI GenBank database under accession codes, OR793334 and OR793335, respectively. The two isolates were deposited in the Culture Collection Ain Shams University (https://ccinfo.wdcm.org/collection/by_id/1186) under the accession number, *B. velezensis* CCASU-C6 and *B. subtilis* isolate CCASU-C11.

### Factors affecting metabolite production

#### Incubation time

**Fig. 2:**
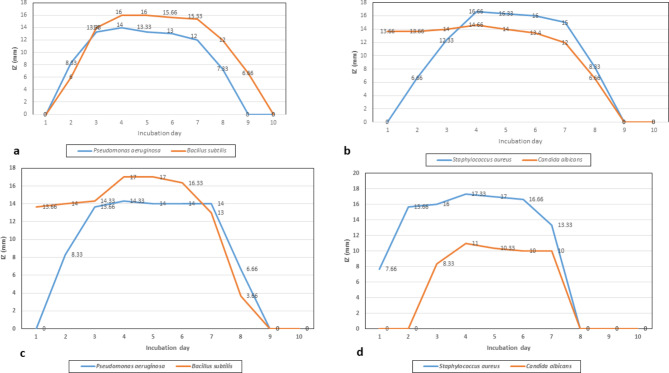
Time course of bioactive metabolite production by (a) *B. velezensis CCASU-C6* against *P. aeruginosa and B. subtilis*, and (b) *B. velezensis CCASU-C6* against *S. aureus* and C. *albicans* (c) *B. subtilis CCASU-C11* against *P. aeruginosa and B. subtilis*, and (d) *B. subtilis* CCASU-C11 isolate against *S. aureus* and C. *albicans*

As shown in Fig. 2, the optimum incubation time for maximum bioactive metabolite production was at 4 days for both *B. velezensis* CCASU-C6 (Fig. 2a & b), and *B. subtilis* CCASU-C11 (Fig. 2c & d). Therefore, 4 days incubation time was fixed for the next experiments.

#### Carbon and nitrogen sources

**Fig. 3:**
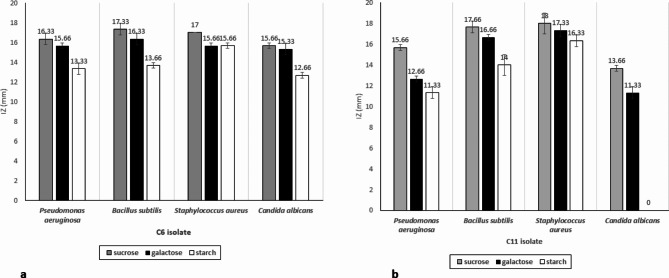
Effect of different carbon sources on bioactive metabolite production from (a) *B. velezensis CCASU-C6*, (b) *B. subtilis CCASU-C11*

**Fig. 4:**
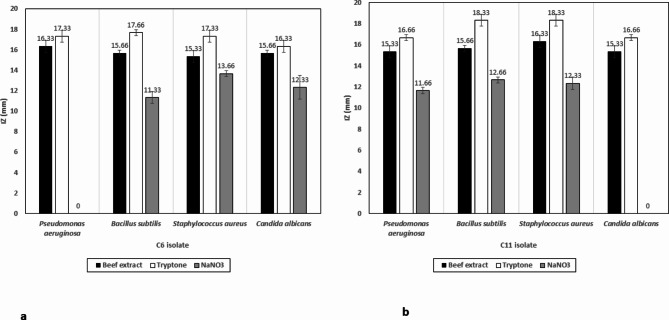
Effect of nitrogen sources on bioactive metabolite production from (a) *B. velezensis CCASU-C6*, and (b) *B. subtilis CCASU-C11*

As shown in Figs. 3 and 4, sucrose and tryptone were found to be the optimum carbon and nitrogen sources for *B. velezensis* CCASU-C6, and *B. subtilis* CCASU-C11, respectively.

#### Effect of environmental conditions on production

The results attained after performing the 16 runs suggested by the design were documented in Table 2 for the isolates C6 and C11. As shown in ANOVA (Table S1), a Model F value of 36.21 and 75.16 was obtained for *B. velezensis* CCAUS-C6, and *B. subtilis* CCASU-C11, respectively, which confirms the models’ significance, since there is only a 0.01% possibility that noise could cause F values this large (*P-value* < 0.0001). Of the tested factors for C6, A, A^2,^ and B^2^ were found to be significant, while for C11, C, B^2^, and C^2^ were significant, with a *P-value* < 0.05 (Table S1). The coefficient of determination, R^2^, was 0.96 for *B. velezensis* CCASU- C6, and 0.98 *B. subtilis* CCASU-C11, meaning that our models can explain 96% and 98% of the response variability, respectively. The Predicted R-squared (Pred R2 = 0.89 for C6, 0.93 for *B. velezensis* CCASU-C6) and Adjusted R-squared (Adj R2 = 0.93 for C6, 0.97 for and *B. subtilis* CCASU-C11) were in acceptable agreement for both isolates. Lastly, the Adequate precision ratio (Adeq prec = 14.61 for *B. velezensis* CCASU-C6, and 22.14 for *B. subtilis* CCASU-C11 showed a reasonable signal and that our models may well be used to navigate the design space.

**Table 2:** Central composite design showing the experiments and the observed responses for *B. velezensis CCASU-C6* metabolite against *B. subtilis* and *B. subtilis* strain C11 metabolite against *C. Albicans*

Run no	Temperature (A)	pH (B)	Agitation rate (C)	Observed response (average IZ, mm)
*B. velezensis* CCASU-C6				
1	33.00	8.00	200.00	14.66
2	33.00	7.00	250.00	13.66
3	37.00	7.00	200.00	17.66
4	29.00	6.00	250.00	0
5	37.00	6.00	250.00	13.66
6	37.00	8.00	250.00	11.33
7	29.00	7.00	200.00	0
8	37.00	8.00	150.00	11.33
9	29.00	8.00	250.00	0
10	33.00	7.00	200.00	19.33
11	29.00	8.00	150.00	0
12	33.00	7.00	200.00	18.66
13	33.00	6.00	200.00	12.33
14	33.00	7.00	200.00	18.66
15	29.00	6.00	150.00	0
16	33.00	7.00	150.00	17.66
*B. subtilis* strain C11				
1	29.00	8.00	150.00	0
2	29.00	7.00	200.00	15.33
3	37.00	8.00	250.00	13.66
4	29.00	6.00	250.00	11.66
5	33.00	7.00	200.00	17.33
6	29.00	6.00	150.00	0
7	33.00	7.00	200.00	17.66
8	37.00	8.00	150.00	0
9	33.00	6.00	200.00	12.0
10	37.00	7.00	200.00	15.33
11	37.00	6.00	150.00	0
12	37.00	6.00	250.00	17.66
13	33.00	7.00	200.00	16.33
14	33.00	7.00	250.00	17.0
15	33.00	8.00	200.00	11.33
16	29.00	8.00	250.00	12.33

From these results, the software automatically projected a model relating the IZ produced with the examined factors. This equation is given as follows: Eq. 1 for *B. velezensis* CCASU-C6:

$$ \begin{aligned} & {\text{IZ }}\left( {{\text{mm}}} \right) = - {771}.{71} + {35}.{53}*{\text{A}} + {5}0.{75}*{\text{B}} - {9}.{84}*{1}0^{{ - {3}}} *{\text{C}} \\ & \quad - 0.{17}*{\text{A}}*{\text{B }} - \, 0.{49}*{\text{ A}}^{{2}} - { 3}.{24 }*{\text{ B}}^{{2}} \\ \end{aligned} $$

Equation 2 for *B. subtilis* strain C11:

$$ \begin{aligned} & {\text{IZ}} = \, - {272}.{45 } - 0.{73 }*{\text{ A }} + {58}.00*{\text{ B }} + 0.{83 }*{\text{ C }} \\ & \quad + {4}.{5}*{ 1}0^{{ - {3}}} *{\text{ A }}*{\text{ C }} \\ & \quad - {4}.{17 }*{\text{ B}}^{{2}} - {2}.{1}0*{ 1}0^{{ - {3}}} *{\text{ C}}^{{2}} \\ \end{aligned} $$

**Fig. 5:**
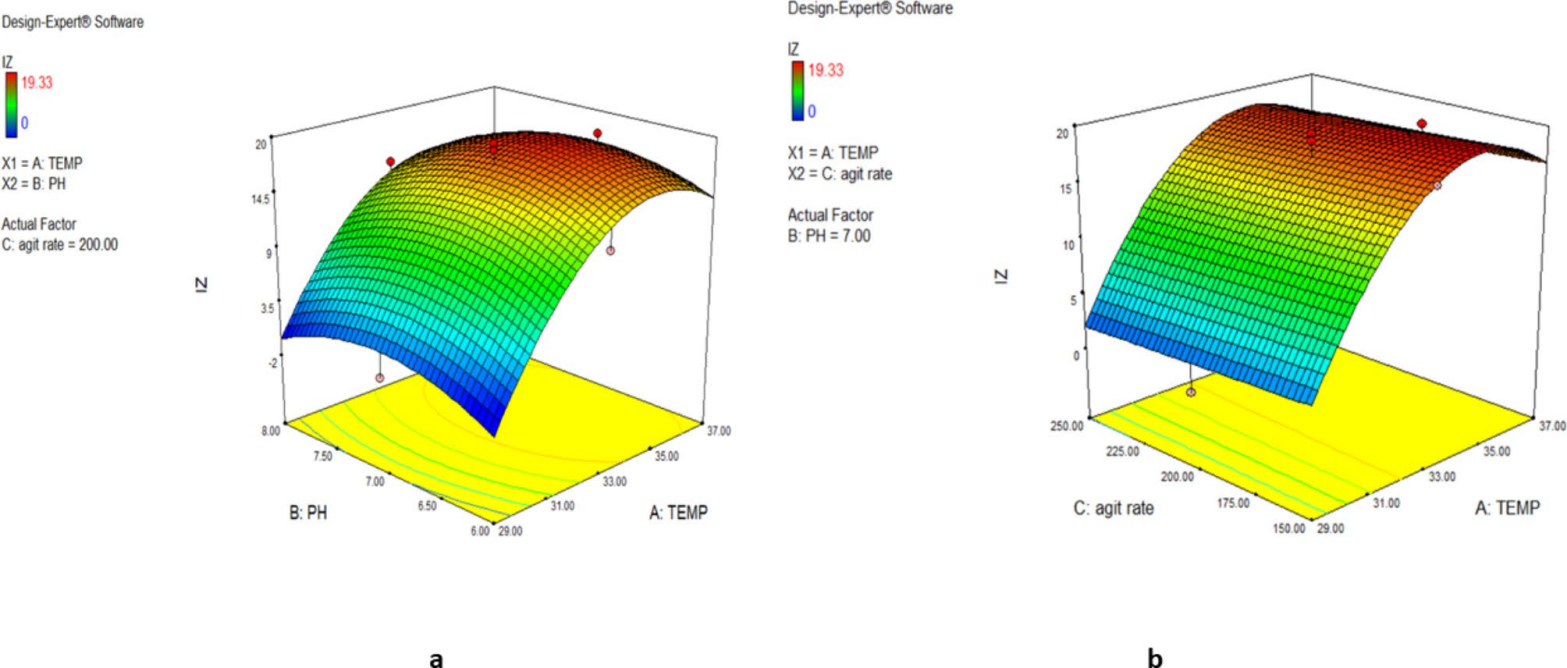
Three-dimensional surface plots representing the effect of 3 factors on metabolite production from *B. velezensis* CCASU-C6. When the effect of two parameters was plotted, the remaining one was set at central level (a) temperature and pH (b) temperature and agitation rate

**Fig. 6:**
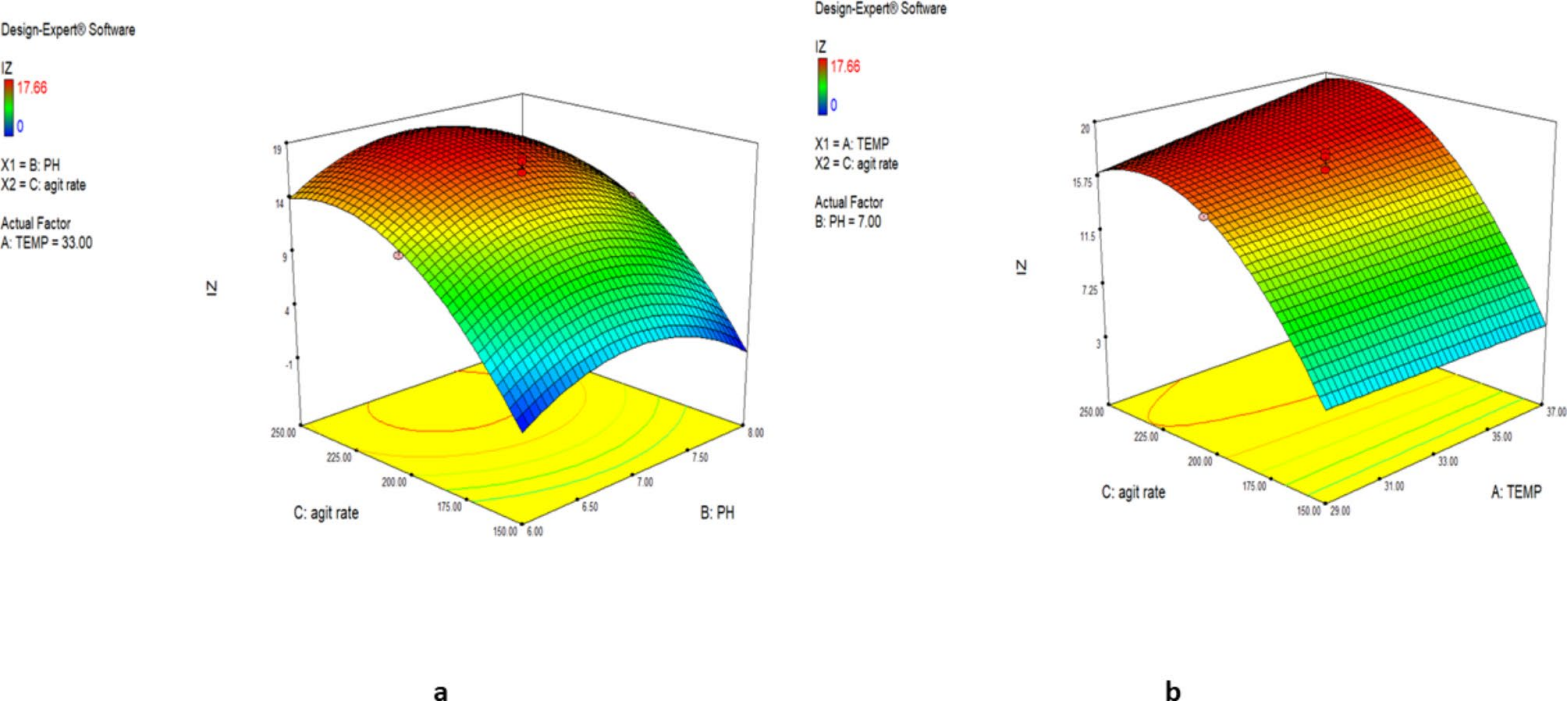
Three-dimensional surface plots representing the effect of 3 factors on metabolite production from *B. subtilis* CCASU-C11. When the effect of two parameters was plotted, the remaining one was set at central level (a) agitation rate and pH (b) agitation rate and temperature

The three-dimensional (3D) plots are shown in Figs. 5 and 6. Utilizing these plots alongside the numerical optimization function in the software, the optimal conditions for highest metabolites production were found to be a pH of 7, temperature of 33 °C and agitation rate of 200 rpm for *B. velezensis* CCASU-C6 metabolite resulting in maximum IZ of 19.33 mm and temperature of 37 °C, pH of 7 and agitation rate of 250 rpm for and *B. subtilis* CCASU-C11 metabolite resulting in a maximum IZ of 19.44 mm.

## *Diagnostic plots*

The results of these plots are depicted in Figures S2, S3 and S4.

Therefore, the optimal conditions generated resulted in a 1.2-fold improvement in metabolite production for *B. velezensis* CCASU-C6 and 1.8-fold improvement in metabolite production for *B. subtilis* CCASU-C11 when contrasted with that produced using unoptimized conditions, as shown in Fig. S4.

## Antioxidant assay

**Fig. 7:**
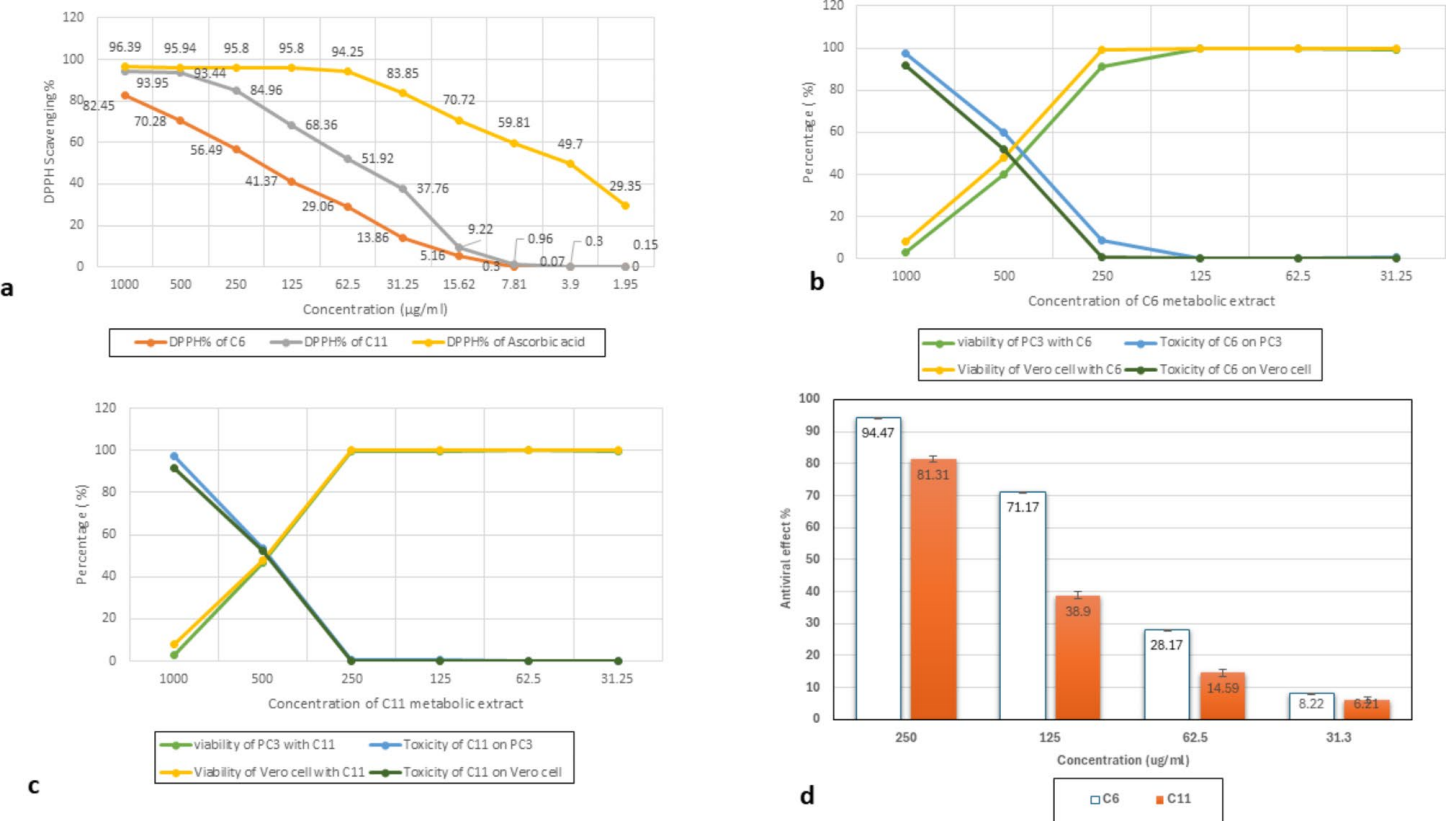
(a) Free radical scavenging activity of crude extracts of *B. velezensis* CCASU-C6 and *B. subtilis* CCASU-C11 in comparison to the control (b) effect of *B. velezensis* CCASU-C6 metabolic extract on Vero cells and PC3 cells, (c) effect of *B. subtilis* CCASU-C11 metabolic extract on Vero cells and PC3 cells (d) Antiviral effects of different concentrations of *B. velezensis* CCASU-C6 and *B. subtilis* CCASU-C11extracts

The DPPH method was utilized to assess the free radical scavenging activity of crude extracts of *B. velezensis* CCASU-C6, and *B. subtilis* CCASU-C11 in comparison to the control (ascorbic acid). The obtained results are documented in Table S2. The maximum DPPH-scavenging activity for ascorbic acid, C6, and C11 isolates was recorded at 1000 µg/mL with percentages of 96.39%, 82.45%, and 93.95%, respectively. The IC50 value represents the concentration of extract required for inhibiting 50% of free radicals. The extract’s antioxidant effectiveness increases as the IC50 value decreases. The IC50% for ascorbic acid, *B. velezensis* CCASU-C6, and *B. subtilis* CCASU-C11 were measured as 6.57 ± 0.17, 199.2 ± 6.77, and 53.76 ± 0.39 µg/mL, respectively (Fig. 7a). This suggests that *B. velezensis* CCASU-C6 crude extract required a higher concentration for scavenging 50% of free radicals than *B. subtilis* CCASU-C11 crude extract. Hence, both crude extracts possessed antioxidant activity, however, the antioxidant activity of the crude extract of *B. subtilis* CCASU-C11 was greater than that of *B. velezensis* CCASU-C6.

### Cytotoxic assay

The obtained data on the cytotoxic activity of the crude extract for both isolates on Vero cells were shown in Table S3 and Fig. S5, and the results of cytotoxic activity against PC3 cells were shown in Table S4 and Fig S5. The 50% cytotoxic concentration (IC50) was evaluated to ensure that the extract was not hazardous to normal cells while also being toxic to cancer cells. From the obtained data, the IC50% of *B. velezensis* CCASU-C6 extract and *B. subtilis* CCASU-C11 extract on the Vero cell was 598.89 ± 4.11 and 599.52 ± 16.03 µg/mL, while on the PC3 cells was 540.27 ± 5.38 and 581.32 ± 4.12 µg/mL, respectively (Fig. 7b). The cytotoxic activity of the extract of C6 and C11 isolates at a concentration of 1000 µg/mL was nearly 91% for both isolates on Vero cells and approximately 97% on cancer cells (Fig. 7c). At a dosage of 500 µg/mL, the cytotoxicity percentage against Vero and cancer cells was nearly 52% for both isolates on Vero cells, approximately 59% for *B. velezensis* CCASU-C6, and 53% for *B. subtilis* CCASU-C11 on cancer cells. At a dosage of 250 ug/ml, the cell viability of Vero and cancer cells remained robust, with minimal toxicity on the cells. Unfortunately, the cytotoxic activity of both isolates was extremely modest. This means that at high doses, the cytotoxic activity was strong on both normal and cancer cells, whereas at low concentrations, toxicity was low on both cells.

#### Antiviral assay

In the present study, the MTT method was utilized to evaluate the antiviral activity of *B. velezensis* CCASU-C6 and *B. subtilis* CCASU-C11 extracts against Vero cells and viral cells (HSV1). As previously mentioned in the cytotoxicity assay, assessing toxicity against Vero cells is critical to ensure the safety of this metabolite during therapy. *B. velezensis* CCASU-C6 and *B. subtilis* CCASU-C11 samples had maximum nontoxic concentrations (MNTCs) at 250 µg/mL. At this concentration, *B. velezensis* CCASU-C6 *and B. subtilis* CCASU-C11 had antiviral efficacy against HSV1 cells of about 94% and 81%, respectively, while Vero cell viability was around 97% and 89%, respectively. At 125 µg/mL, the antiviral activity of C6 on the viral cells was 71%, and cell viability remained high, whereas the antiviral activity of the *B. subtilis* CCASU-C11 sample on the viral cells at the same concentration was approximately 39%, and cell viability decreased. At 62.5 µg/mL or below, the antiviral efficacy was weak, viral activity was high, and cell viability was reduced. This suggests that both isolates displayed antiviral activity, especially at concentrations of 250 µg/mL; however, the antiviral activity of *B. velezensis* CCASU-C6 was higher than that of *B. subtilis* CCASU-C11. Table S5 and Fig. 7d show the results of the antiviral activity of *B. velezensis CCASU-C6 and B. subtilis* CCASU-C11 samples.

### Genomic analysis for identification of the biosynthetic gene clusters

**Fig. 8:**
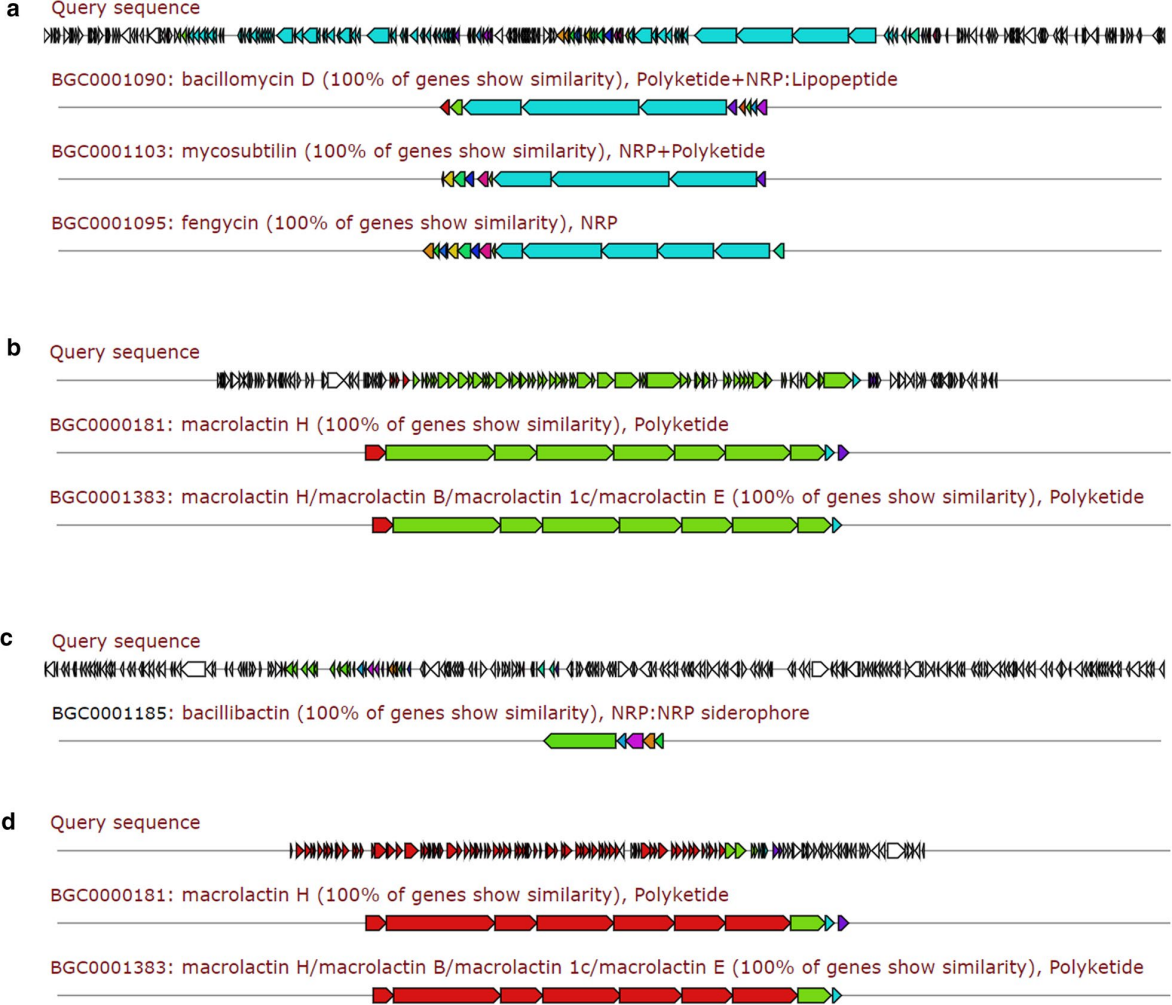
Conserved biosynthetic gene clusters in *B. velezensis* CCASU-C6 of bacillomycin (a), mycosubtilin, fengycin (a; ORFs in blue color indicates conserved biosynthetic genes, and gene cluster for Macrolactin H (b, ORFs in green color indicates conserved biosynthetic genes) and in *B. subtilis* CCASU-C11 of bacillibactin (c; ORFs in green color indicates conserved biosynthetic genes) and gene cluster for Macrolactin H (d; ORFs in red color indicates conserved biosynthetic genes)

The secondary metabolite gene analysis using antiSMASH software revealed that:

For *B. velezensis* CCASU-C6, it showed 100% similarity of the biosynthetic gene clusters of:

- Bacillomycin (antifungal polypeptide antibiotic) (Fig. 8a).

b) Mycosubtilin (natural lipopeptide with antifungal and hemolytic activities) (Fig. 8a).
c) Fengycin (a cyclic lipopeptide with fungicidal activity against many plant and animal fungal pathogens) (Fig. 8a).
d) Macrolactin H (macrolides with excellent broad-spectrum antibacterial activity) (Fig. 8b).

For *B. subtilis* CCASU-C11, it showed 100% similarity of the biosynthetic gene clusters of:

- Bacillibactin (catechol-based siderophore having antimicrobial activity) (Fig. 8c).
- Macrolactin H (macrolides with excellent broad-spectrum antibacterial activity) (Fig. 8d).

## Discussion

The existing antibiotic resistance among different harmful bacteria necessitates alternative therapies (Mengoni et al. 2014). In this study, 19 endophytic bacterial isolates were obtained from garlic tissues such as cloves, roots, and leaves collected from Munufia in Cairo for endophytic bacterial isolation. Upon preliminary identification of the isolated endophytic bacteria, all isolates were found to be Gram-positive bacilli. Screening was then carried out to select those isolates with potent antagonistic effects against different human bacterial and fungal pathogens. Out of 19 bacterial isolates, 12 exhibited antibacterial effectiveness against the standard pathogenic species (*B. subtilis*,* P. aeruginosa*,* S. aureus*), and five isolates exhibited antifungal activity against *C. albicans.* To the best of our knowledge, this is the first research that presents the antibacterial activity of metabolites produced by endophytic bacteria isolated from garlic plant. In 1995, Susilowati et al. demonstrated that *B. subtilis* products can inhibit harmful bacteria such as MRSA and other *Staphylococcus sp*. (Susilowati et al. 2015). Chao et al. discovered antifungal metabolites in *B. amyloliquefaciens* isolated from Cinnamomum camphora leaves(Lin et al. 2020). Yuan et al. proved the anti-fungal efficacy of metabolic extract produced by *B. amyloliquefaciens* isolated from the *Ginkgo biloba* (Yuan et al. 2012). Wahjono et al. also discovered the antibacterial activity of the crude extract of endophytic bacteria isolated from seagrass *Enhalus* sp. and belonging to *Bacillus sp*. (Sulistiyani et al. 2015).

The two isolates, C6 and C11, showed broad-spectrum activity and were hence selected for further studies. The selected bacterial isolates, C6 and C11, were identified molecularly using 16 S rRNA gene sequencing. A phylogenetic tree of the 16 S ribosomal RNA gene sequence was built using MEGA X, and it revealed that the C6 isolate clustered near the *B. velezensis* strain and the C11 isolate near *B. subtilis subsp. subtilis*. Accordingly, C6 and C11 isolates were identified as *B. velezensis* CCASU-C6, and *B. subtilis* CCASU-C11, respectively. The incubation period, carbon and nitrogen content of the culture medium, and environmental circumstances can impact the production of bioactive metabolites as previously reported (Webster and Shepherd 2023; Fracchia-Durán et al. 2023). This study aimed to optimize nutritional and environmental conditions for the endophytic bacterial isolate to produce maximum antimicrobial metabolites.

The incubation period and different carbon and nitrogen sources of the production media were studied using OFAT (One Factor at a Time) technique. At the end of each run, the antimicrobial activity of the C6 and C11 endophytic bacterial isolates was assessed against standard isolates (*B. subtilis*,* P. aeruginosa*,* S. aureus*, and *C. albicans*). As shown in the results, an incubation time of 4 days, sucrose, and tryptone were selected to be the optimum conditions for maximum antimicrobial activity. In a similar study, it was proven that when multiple carbon sources were supplemented to the fermentation medium, sucrose was the best carbon source to create maximal metabolite production from endophytic bacteria(Beltran-Gracia et al. 2017). Liu X et al. also found that sucrose was the ideal carbon source for increasing the production and antifungal activity of the metabolite (surfactin) from *B. amyloliquefaciens* (Liu et al. 2012). Anjum and Chandra discovered that tryptone was the ideal nitrogen source for the fermentation medium to produce metabolites from *Catharanthus roseus* (Anjum and Chandra 2019).

Other factors including incubation temperature, pH of the production media, and agitation rate were optimized using Response surface methodology which is a blend of mathematical and statistical tactics for describing the behavior of a data set and making statistical predictions. It is beneficial when numerous variables impact a response or a set of related responses of interest. The objective is to achieve optimal system performance (Bezerra et al. 2008). This work aimed to optimize the synthesis of antimicrobial metabolites by the C6 and C11 isolates through a central composite design. After each run, the antibacterial activity of the C6 endophytic bacterial isolate was assessed against *B. subtilis* ATCC 6633, while the antifungal activity of the *B. subtilis* CCASU-C11 bacterial isolate was evaluated against *C. albicans* ATCC10231. After performing the 16 proposed runs, a significant model was obtained as proved by the ANOVA analysis.

The 3D response surface curves with three variables generated by the software were used to demonstrate the effect of two parameters at a time while maintaining the third constant. As a consequence, these charts were more beneficial in understanding the interaction effects of these two factors (Abhini et al. 2022) and allowing for easy calculation of the ideal experimental conditions (Ibrahim et al. 2019). Based on these graphs, and the numerical optimization method, the optimum conditions for the C6 isolate were incubation temperature at 33 °C, pH of the production media at 7, and agitation rate at 200 rpm, resulting in a maximal IZ of 19.33 mm, while the ideal conditions for C11 isolate were found to be at the incubation temperature at 37 °C, pH of 7, and agitation rate at 250 rpm to produce maximal IZ of 19.44 mm.

Temperature influences the outcome of fermentation reactions. It regulates the growth of bacteria and metabolite production, and it varies according to the type of organism (Banerjee and Bhattacharyya 1992). The antimicrobial metabolites production was found to be maximum at 33 °C for the C6 isolate and 37 °C for the C11 isolate. A previous study suggested the optimum temperature to yield the maximum production of metabolites from endophytic bacteria was 33 °C (Anjum and Chandra 2019). Another factor that influences the bacterial growth and synthesis of metabolites is pH. From the obtained results, both isolates showed maximum production of antimicrobial metabolites at pH 7. Furthermore, the agitation rate is a crucial factor that influences antimicrobial metabolite production by providing appropriate mixing, enhancing oxygen transfer, and preserving uniform physical and chemical properties in the fermentation media. The ideal agitation rate for maximum antimicrobial metabolite production from the C6 isolate was 200 rpm, while the optimal agitation speed for maximum metabolite production from the C11 isolation was 250 rpm.

ANOVA was used to analyze the results, verify model applicability, and identify factors affecting antimicrobial metabolite production. The P-value determines the significance of the results (Ibrahim et al. 2019). ANOVA results proved that the model equations obtained were able to accurately describe metabolite production for both isolates. In this study, a graphical summary of case statistics was created to validate the models. The Box-Cox plot technique simplifies data transformation and reduces the heterogeneity of errors (Atkinson 1986). The Box-Cox data transformation is an easy method for analyzing non-normal data sets. The present lambda value is inside the 95% confidence range; hence no transformation is recommended. The predicted versus actual plot is a quantitative comparison of the experimental response values to the predicted values from the developed models. The residual versus run plot displays the residuals in relation to the experimental run order (Ibrahim et al. 2019). The plots created in this study validate our models.

In the present study, the crude extracts of C6 and C11 were used to evaluate the antioxidant (free radical scavenging activity), anticancer, and antiviral effectiveness. The crude extracts’ free radical scavenging effects showed significant antioxidant capacity when compared to ascorbic acid. The DPPH scavenging was high because the test was performed at high concentrations ranging from 1000 µg/mL to 500 µg/mL for C6 crude extract and 1000 µg/mL to 125 µg/mL for C11 extract. The DPPH scavenging% became low when the concentration of the crude extract of C6 became lower than 500 µg/mL, whereas the scavenging became low when the concentration of the C11 crude extract was lower than 125 µg/mL. This is a strong indication that the C6 and C11 endophytes can be a valuable source of bioactive compounds for the development of innovative medicines. Akinsanya et al. found that the crude extract obtained from bacterial endophytes isolated from *Aloe Vera* had antioxidant activity, attributed to flavonoids and alkaloid compounds in the extract (Akinsanya et al. 2015). Furthermore, previous studies reported that the presence of bioactive substances such as polyphenolic compounds and flavonoids is responsible for the antioxidant activity of the plant extract (Falcioni et al. 2002; Aini et al. 2022). Similar research has indicated that *Phyllosticta* sp. isolated from *Guazuma tomentosa*, had potent antioxidant properties due to the existence of bioactive substances in their metabolites (Perez Gutierrez and Neira González 2019).

Regarding the cytotoxic activity assay, which was performed using the MTT method against the PC3 cancer cell and the normal Vero cell, the results showed that the C6 and C11 extracts showed modest cytotoxic activity and were not suitable for use in cancer treatment. A previous similar study demonstrated that the extract of endophytic bacteria isolated from *Solanum torvum* Sw (*Solanaceae*) had cytotoxicity on healthy cells ((Christian et al. 2022) Another study examined the cytotoxic efficacy of ethanol metabolic extracts derived from *Andalas* endophytic bacteria on Vero cells, and the results showed that the extract may kill the cell at high concentrations, and it was not recommended for use in cancer treatment(Putri et al. 2023).

Viruses pose a global hazard to human health and safety. So, discovering novel sources with high efficacy and low side effects for viral illness treatment is critical. In the current study, results revealed that the extracts of C6 and C11 had high antiviral efficacy against the HSV1 virus while did not affect normal Vero cells. The results showed that the endophytic bacterial strains C6 and C11 are a valuable source of antivirals. Gayathri et al. reported that the metabolic extract of bacterial endophytes has antiviral activity(Gayathri et al. 2024).

For deciphering the nature of the bioactive metabolites, genomic sequencing using ONT analysis of both isolates has been undertaken followed by AntiSMASH analysis to extract the secondary metabolite biosynthetic gene clusters particularly those having antimicrobial activities as previously reported its effectiveness and sensitivity for such purposes (Blin et al. 2021).

Our findings revealed the conservation of 100% of biosynthetic gene clusters of four antimicrobial metabolites in *B. velezensis* CCASU-C6, and two antimicrobial metabolites in *B. subtilis* CCASU-C11. The antimicrobial metabolites of *B. velezensis* CCASU-C6, were bacillomycin (antifungal polypeptide antibiotic) (Peypoux et al. 1981), mycosubtilin (natural lipopeptide with antifungal and hemolytic activities (Besson and Michel 1989), fengycin (cyclic lipopeptide with fungicidal activity against many plant and animal fungal pathogens (Lin et al. 2020), (Geissler et al. 2019) and macrolactin H (macrolides with excellent broad spectrum antibacterial activity (Wu et al. 2021) which is also was found in *B. subtilis* CCASU-C11 in addition to Bacillibactin which is a catechol-based siderophore with antimicrobial activity (Chakraborty et al. 2022). Both bacillomycin and mycosubtilin belong to the iturin lipopeptide family and are commonly produced by *Bacillus* species, as previously reported (Peypoux et al. 1981; Besson and Michel 1989). However, the respective identified bioactive metabolites are first reported in this study to be collectively produced by the identified endophytic strains.

Fengycin exhibits strong antifungal activity against many animal and plant pathogens, and its production was previously reported by many *Bacillus* and *Alcaligenes* species (Eltokhy et al. 2024). The results of the genomic analysis and detection of the respective biosynthetic gene clusters confirmed the antimicrobial activities of the recovered endophytic isolates. However, scaling up the production, isolation in pure form, and clinical evaluation of the respective bioactive metabolites are strongly advised as a future perspective of this work and for evaluating their potential use in humans.

In this study, two promising endophytic bacteria namely, *B. velezensis* CCASU-C6, and *B. subtilis* CCASU-C11 were isolated from garlic plants and produced various metabolites with broad-spectrum antibacterial, antifungal activity, antioxidant, and antiviral activities. A successful statistical model was created for optimizing various environmental and growth conditions and resulted in increasing production of the antibacterial activities of *B. velezensis* CCASU-C6 and *B. subtilis* CCASU-C11 by 1.2 and 1.8-fold, respectively. Genomic analysis for identification of the biosynthetic gene clusters of the produced antimicrobial metabolites showed 100% conservation in the gene clusters of bacillomycin, mycosubtilin, fengycin, macrolactin H in *B. velezensis* CCASU-C6 and bacillibactin and macrolactin H in *B. subtilis* strain C1. Scaling up the production of the respective metabolites in a fermenter is highly recommended for industrial production of the respective bioactive metabolites in large quantities and in pure forms to be suitable for drug development.

## Electronic supplementary material

Below is the link to the electronic supplementary material.

Supplementary Material 1

## Data Availability

and Material. This published article and supplementary file include all data generated, and, or analyzed during this present study. The 16 S ribosomal RNA nucleotide sequences included in this study were submitted to the NCBI GenBank database under accession codes, OR793334 and OR793335, respectively.
