# Supplementary material for: Deciphering the nature and statistical optimization of antimicrobial metabolites of two endophytic bacilli
Source: AMB Express. 2025 Jan 13;15:10. doi: 10.1186/s13568-024-01811-3 (PMC11730024; doi:10.1186/s13568-024-01811-3)
Supplement: Supplementary file 1 — Supplementary Material 1 [file 13568_2024_1811_MOESM1_ESM.docx]

**Table S1.** ANOVA for Response Surface Reduced Quadratic Model for metabolites of C6 and C11 metabolites

| **C6 Metabolite** | | | | | |
| --- | --- | --- | --- | --- | --- |
| **Source** | **Sum of quares** | **Degrees of freedom** | **Mean Square** | **F Value** | **P - value** |
| Model | 870.80 | 6 | 145.13 | 36.21 | < 0.0001 |
| A-TEMP | 386.36 | 1 | 386.36 | 96.40 | < 0.0001 |
| B-PH | 0.87 | 1 | 0.87 | 0.22 | 0.6529 |
| C-agit rate | 1.99 | 1 | 1.99 | 0.50 | 0.4991 |
| AB | 3.06 | 1 | 3.06 | 0.76 | 0.4049 |
| A2 | 183.86 | 1 | 183.86 | 45.88 | < 0.0001 |
| B2 | 30.86 | 1 | 30.86 | 7.70 | 0.0216 |
| Residual | 36.07 | 9 | 4.01 |  |  |
| Lack of Fit | 35.77 | 7 | 5.11 | 34.15 |  |
| Pure Error | 0.30 | 2 | 0.15 |  |  |
| Cor Total | 906.87 | 15 |  |  |  |
| **C11 Metabolite** | | | | | |
| **Source** | **Sum of Squares** | **Degrees of freeedom** | **Mean Square** | **F Value** | **P- value** |
| Model | 710.73 | 6 | 118.45 | 75.16 | < 0.0001 |
| A-TEMP | 5.37 | 1 | 5.37 | 3.41 | 0.0979 |
| B-PH | 1.60 | 1 | 1.60 | 1.02 | 0.3400 |
| C-agit rate | 396.87 | 1 | 396.87 | 251.80 | < 0.0001 |
| AC | 6.72 | 1 | 6.72 | 4.26 | 0.0690 |
| B2 | 38.78 | 1 | 38.78 | 24.61 | 0.0008 |
| C2 | 64.92 | 1 | 64.92 | 41.19 | 0.0001 |
| Residual | 14.18 | 9 | 1.58 |  |  |
| Lack of Fit | 13.23 | 7 | 1.89 | 3.94 | 0.2174 |
| Pure Error | 0.96 | 2 | 0.48 |  |  |
| Cor Total | 724.91 | 15 |  |  |  |

**Table S2.** Free radical scavenging activity of crude extracts of C6 and C11 isolates in comparison to the control

| **Code** | **Conc (µg/ml)** | **Absorbance** | | | **Mean** | **SE** | **DPPH scavenging %** | **IC50 ± SD** |
| --- | --- | --- | --- | --- | --- | --- | --- | --- |
|  |  | **1** | **2** | **3** |  |  |  |  |
| Control | 0 | 0.452 | 0.449 | 0.455 | 0.452 | 0.001732 | 0 |  |
| Ascorbic acid | 1000 | 0.017 | 0.015 | 0.017 | 0.016 | 0.000667 | 96.389 | 6.57 ± 0.17 |
|  | 500 | 0.019 | 0.017 | 0.019 | 0.018 | 0.000667 | 95.944 |  |
|  | 250 | 0.018 | 0.019 | 0.02 | 0.019 | 0.000577 | 95.796 |  |
|  | 125 | 0.021 | 0.018 | 0.018 | 0.019 | 0.001 | 95.796 |  |
|  | 62.5 | 0.025 | 0.031 | 0.022 | 0.026 | 0.002646 | 94.248 |  |
|  | 31.25 | 0.076 | 0.069 | 0.074 | 0.073 | 0.002082 | 83.850 |  |
|  | 15.62 | 0.143 | 0.128 | 0.126 | 0.132 | 0.005364 | 70.723 |  |
|  | 7.81 | 0.175 | 0.188 | 0.182 | 0.182 | 0.003756 | 59.808 |  |
|  | 3.9 | 0.231 | 0.227 | 0.223 | 0.227 | 0.002309 | 49.779 |  |
|  | 1.95 | 0.315 | 0.322 | 0.321 | 0.319 | 0.002186 | 29.351 |  |
| C6 | 1000 | 0.075 | 0.085 | 0.078 | 0.0793 | 0.002963 | 82.448 | 199.2 ± 6.77 |
|  | 500 | 0.142 | 0.128 | 0.133 | 0.134 | 0.004096 | 70.280 |  |
|  | 250 | 0.203 | 0.198 | 0.189 | 0.197 | 0.004096 | 56.490 |  |
|  | 125 | 0.265 | 0.266 | 0.264 | 0.265 | 0.000577 | 41.372 |  |
|  | 62.5 | 0.314 | 0.326 | 0.322 | 0.321 | 0.003528 | 29.056 |  |
|  | 31.25 | 0.389 | 0.387 | 0.392 | 0.390 | 0.001453 | 13.864 |  |
|  | 15.62 | 0.423 | 0.43 | 0.433 | 0.429 | 0.002963 | 5.162 |  |
|  | 7.81 | 0.447 | 0.454 | 0.451 | 0.451 | 0.002028 | 0.295 |  |
|  | 3.9 | 0.45 | 0.453 | 0.452 | 0.452 | 0.000882 | 0.074 |  |
|  | 1.95 | 0.455 | 0.45 | 0.451 | 0.452 | 0.001528 | 0 |  |
| C11 | 1000 | 0.024 | 0.03 | 0.028 | 0.027 | 0.001764 | 93.953 | 53.76 ± 0.39 |
|  | 500 | 0.032 | 0.028 | 0.029 | 0.030 | 0.001202 | 93.437 |  |
|  | 250 | 0.075 | 0.067 | 0.062 | 0.068 | 0.003786 | 84.956 |  |
|  | 125 | 0.143 | 0.145 | 0.141 | 0.143 | 0.001155 | 68.363 |  |
|  | 62.5 | 0.213 | 0.220 | 0.219 | 0.217 | 0.002186 | 51.917 |  |
|  | 31.25 | 0.289 | 0.276 | 0.279 | 0.281 | 0.00393 | 37.758 |  |
|  | 15.62 | 0.408 | 0.414 | 0.409 | 0.410 | 0.001856 | 9.2183 |  |
|  | 7.81 | 0.446 | 0.449 | 0.448 | 0.448 | 0.000882 | 0.959 |  |
|  | 3.9 | 0.450 | 0.449 | 0.453 | 0.451 | 0.001202 | 0.295 |  |
|  | 1.95 | 0.451 | 0.453 | 0.450 | 0.451 | 0.000882 | 0.147 |  |

**Table S3.** Effect of C6 and C11 extracts on Vero cells at different concentrations

| **ID** | **Conc (µg/ml)** | **Absorbance** | | | **Mean Abs** | **±SE** | **Viability %** | **Toxicity**  **%** | **IC50 ± SD** |
| --- | --- | --- | --- | --- | --- | --- | --- | --- | --- |
| Vero | -------- | 0.771 | 0.764 | 0.766 | 0.767 | 0.002082 | 100 | 0 |  |
| C6 | 1000 | 0.063 | 0.058 | 0.072 | 0.064 | 0.004096 | 8.388 | 91.612 | 598.89 ± 4.11 |
|  | 500 | 0.357 | 0.387 | 0.362 | 0.369 | 0.00928 | 48.066 | 51.934 |  |
|  | 250 | 0.766 | 0.758 | 0.761 | 0.762 | 0.002333 | 99.305 | 0.695 |  |
|  | 125 | 0.763 | 0.77 | 0.759 | 0.764 | 0.003215 | 99.609 | 0.391 |  |
|  | 62.5 | 0.769 | 0.762 | 0.763 | 0.765 | 0.002186 | 99.696 | 0.304 |  |
|  | 31.25 | 0.766 | 0.772 | 0.761 | 0.766 | 0.00318 | 99.913 | 0.087 |  |
| C11 | 1000 | 0.011 | 0.085 | 0.094 | 0.063 | 0.026295 | 8.257 | 91.743 | 599.52 ± 16.03 |
|  | 500 | 0.354 | 0.367 | 0.374 | 0.365 | 0.005859 | 47.588 | 52.412 |  |
|  | 250 | 0.772 | 0.764 | 0.763 | 0.766 | 0.002848 | 99.913 | 0.087 |  |
|  | 125 | 0.768 | 0.77 | 0.761 | 0.766 | 0.002728 | 99.913 | 0.087 |  |
|  | 62.5 | 0.766 | 0.772 | 0.763 | 0.767 | 0.002646 | 100 | 0 |  |
|  | 31.25 | 0.769 | 0.771 | 0.761 | 0.767 | 0.003055 | 100 | 0 |  |

**Table S4**. Effect of C6 and C11 samples on PC3 cells at different concentrations

| **ID** | **Conc. (µg/ml)** | **Absorbance** | | | **Mean Abs** | **±SE** | **Viability %** | **Toxicity %** | **IC50 ± SD** |
| --- | --- | --- | --- | --- | --- | --- | --- | --- | --- |
| PC3 | -------- | 0.638 | 0.641 | 0.644 | 0.641 | 0.001732 | 100 | 0 |  |
| C6 | 1000 | 0.017 | 0.017 | 0.018 | 0.017 | 0.000333 | 2.704 | 97.296 | 540.27 ± 5.38 |
|  | 500 | 0.264 | 0.257 | 0.251 | 0.257 | 0.003756 | 40.146 | 59.854 |  |
|  | 250 | 0.587 | 0.592 | 0.58 | 0.586 | 0.00348 | 91.472 | 8.528 |  |
|  | 125 | 0.647 | 0.633 | 0.638 | 0.639 | 0.004096 | 99.740 | 0.260 |  |
|  | 62.5 | 0.635 | 0.631 | 0.649 | 0.638 | 0.005457 | 99.584 | 0.416 |  |
|  | 31.25 | 0.64 | 0.632 | 0.639 | 0.637 | 0.002517 | 99.376 | 0.624 |  |
| C11 | 1000 | 0.018 | 0.017 | 0.019 | 0.018 | 0.000577 | 2.808 | 97.192 | 581.32 ± 4.12 |
|  | 500 | 0.311 | 0.286 | 0.301 | 0.299 | 0.007265 | 46.698 | 53.302 |  |
|  | 250 | 0.636 | 0.641 | 0.64 | 0.639 | 0.001528 | 99.688 | 0.312 |  |
|  | 125 | 0.644 | 0.634 | 0.637 | 0.638 | 0.002963 | 99.584 | 0.416 |  |
|  | 62.5 | 0.638 | 0.643 | 0.64 | 0.640 | 0.001453 | 99.896 | 0.104 |  |
|  | 31.25 | 0.635 | 0.648 | 0.636 | 0.640 | 0.004177 | 99.792 | 0.208 |  |

**Table S5.** Effect of C6 and C11 samples on viral cells

| **Test** | **Conc. (µg /mL)** | **Absorbance** | | | **Mean Abs** | **±SE** | **Viability%** | **Toxicity%** | **Viral activity %** | **Anti viral effect %** |
| --- | --- | --- | --- | --- | --- | --- | --- | --- | --- | --- |
| Vero |  | 0.744 | 0.738 | 0.747 | 0.743 | 0.00229 | 100 | 0 |  |  |
| HSV1 |  | 0.352 | 0.337 | 0.348 | 0.346 | 0.00388 | 46.523 | 53.477 | 99.914 | 0.086 |
| C6 | 250 | 0.725 | 0.718 | 0.72 | 0.721 | 0.00180 | 97.039 | 2.961 | 5.532 | 94.467 |
|  | 125 | 0.624 | 0.619 | 0.642 | 0.628 | 0.00604 | 84.567 | 15.433 | 28.834 | 71.166 |
|  | 62.5 | 0.473 | 0.457 | 0.442 | 0.457 | 0.00775 | 61.552 | 38.448 | 71.834 | 28.166 |
|  | 31.3 | 0.398 | 0.366 | 0.37 | 0.378 | 0.00871 | 50.875 | 49.125 | 91.783 | 8.217 |
| C11 | 250 | 0.653 | 0.682 | 0.671 | 0.669 | 0.00732 | 89.996 | 10.004 | 18.692 | 81.308 |
|  | 125 | 0.518 | 0.486 | 0.496 | 0.500 | 0.00818 | 67.295 | 32.705 | 61.105 | 38.895 |
|  | 62.5 | 0.392 | 0.415 | 0.403 | 0.403 | 0.00575 | 54.284 | 45.716 | 85.413 | 14.587 |
|  | 31.3 | 0.367 | 0.372 | 0.371 | 0.370 | 0.00132 | 49.798 | 50.202 | 93.795 | 6.2052 |

| Endophyte extract of C6 against: | Endophyte extract of C11 against: |
| --- | --- |
| (a) *P. aeruginosa* ATCC 9022 | |
| 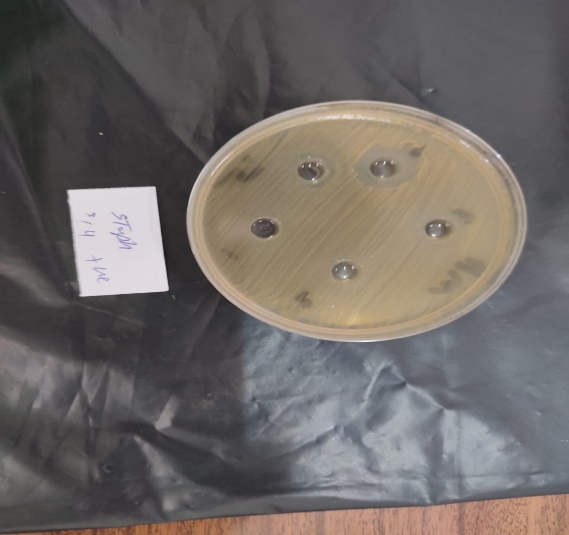 | 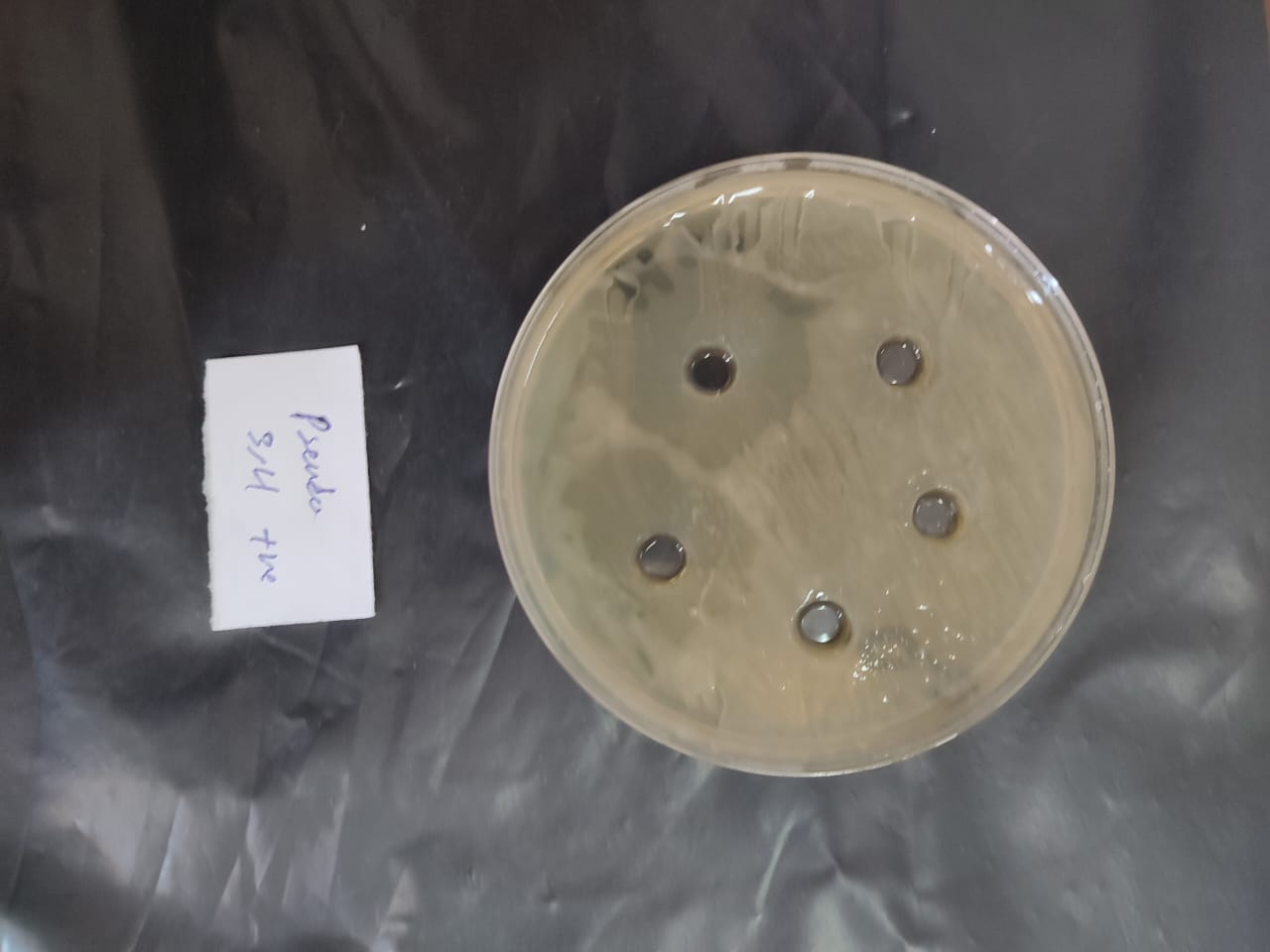 |
| (b) *B. subtilis* ATCC 6633 | |
| 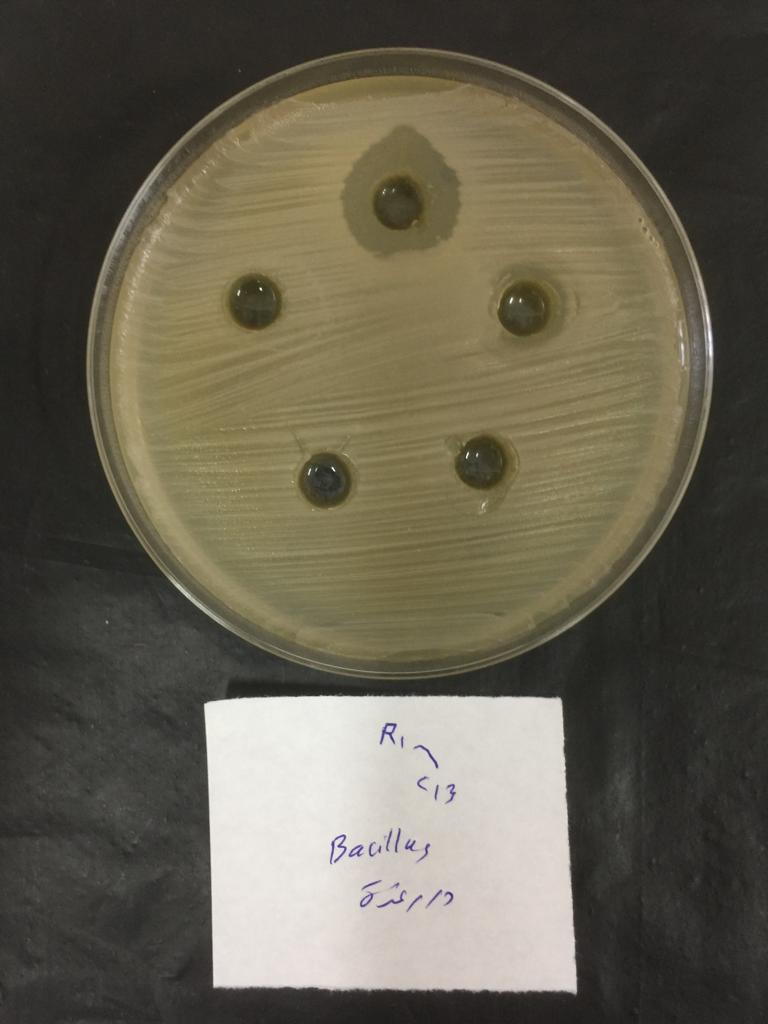 | 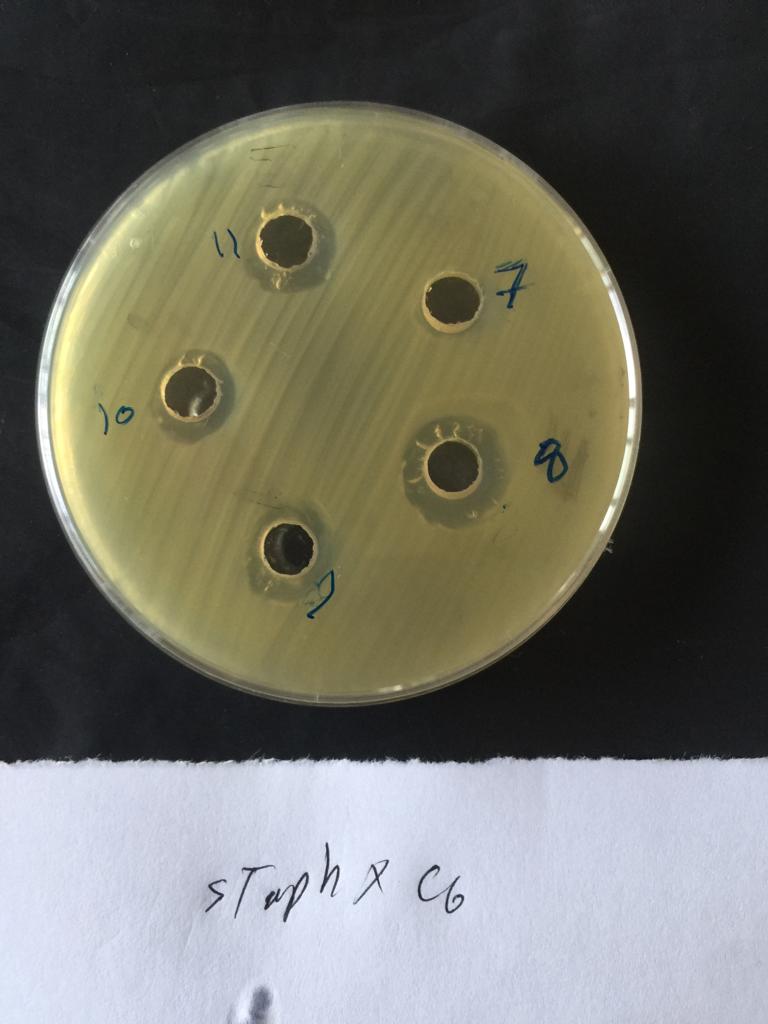 |
| (c) *S. aureus* ATCC 6538 | |
| 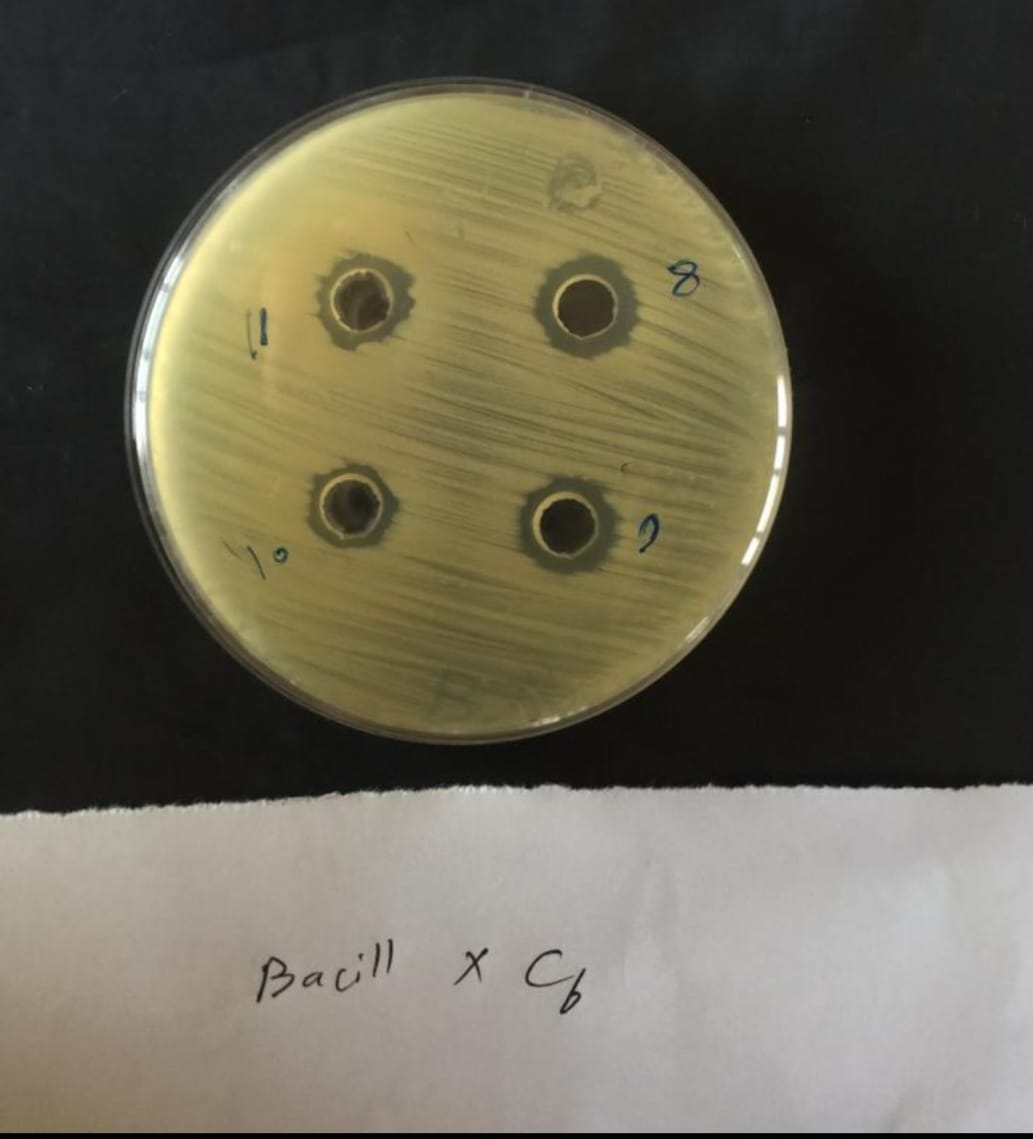 | 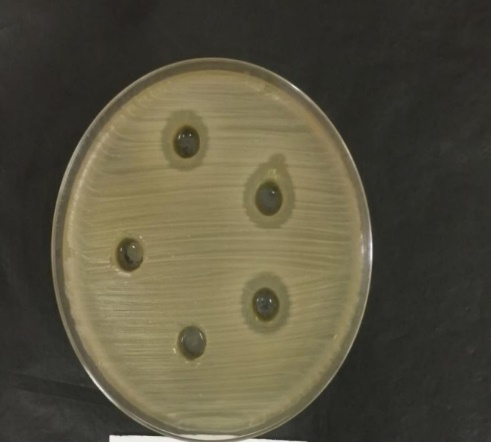 |
| (d) *C. albicans* ATCC10231 | |
| 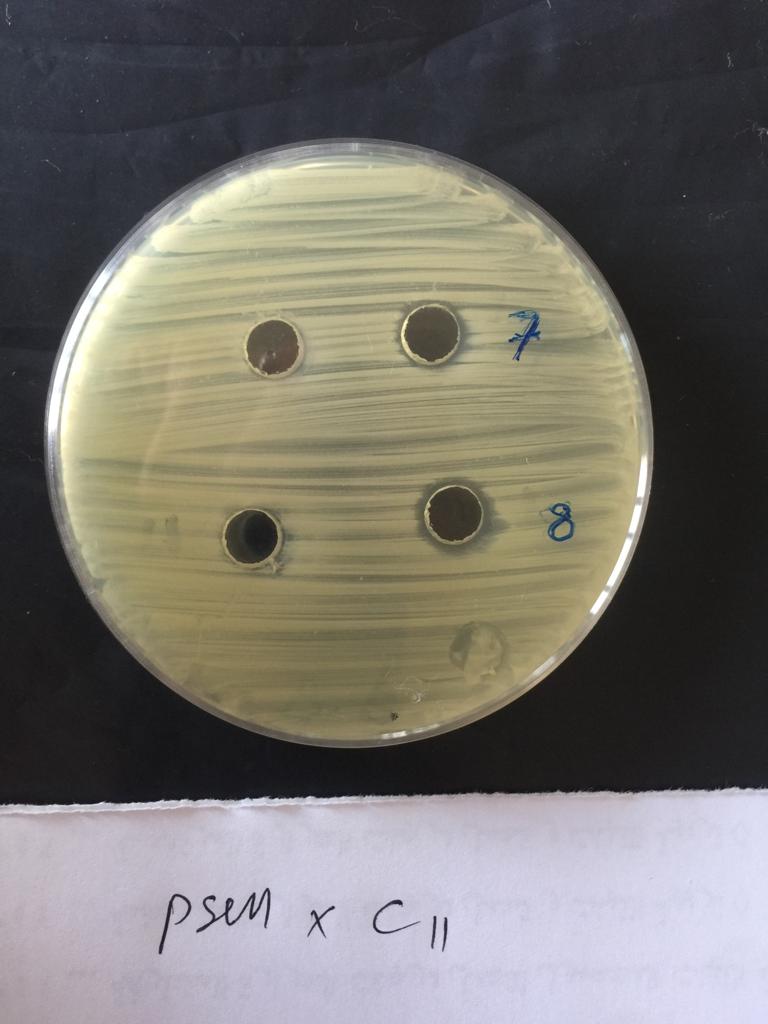 | 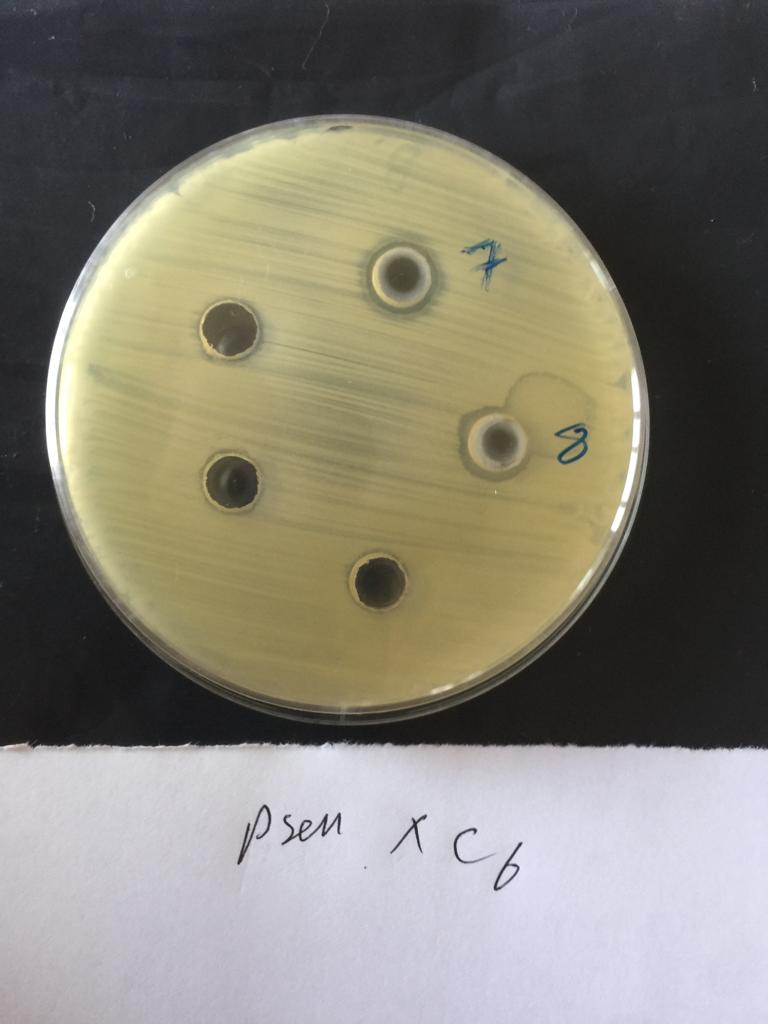 |

**Fig. S1**. Antibacterial activity using cup plate technique of the crude extract of the endophytic isolates C6 and C11 against (a) *P. aeruginosa* ATCC 9022, (b) *B. subtilis* ATCC 6633, (c) *S. aureus* ATCC 6538, (d) *C. albicans* ATCC10231 standard strains.


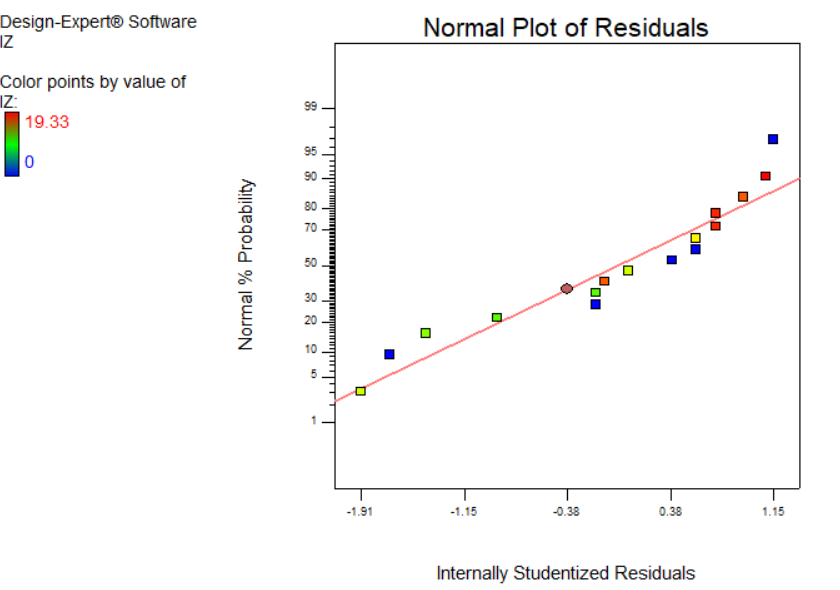


**a**


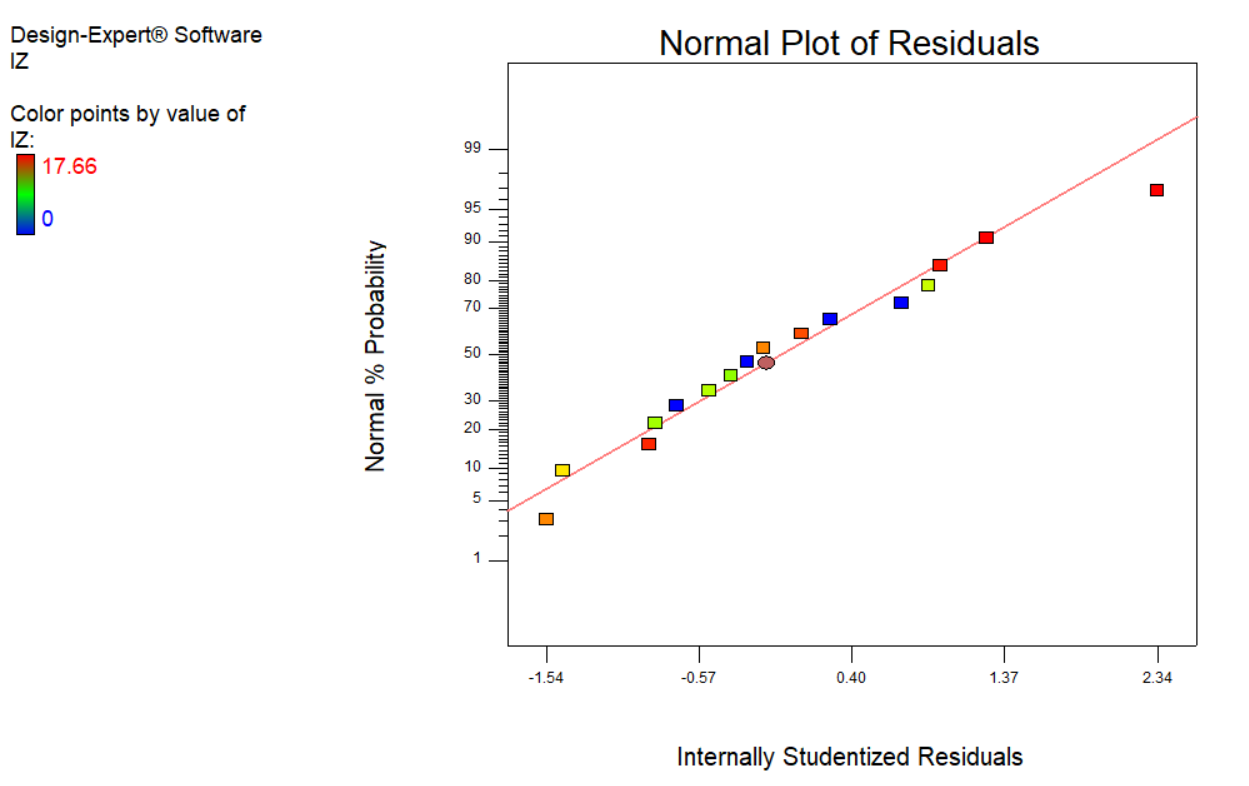


**b**


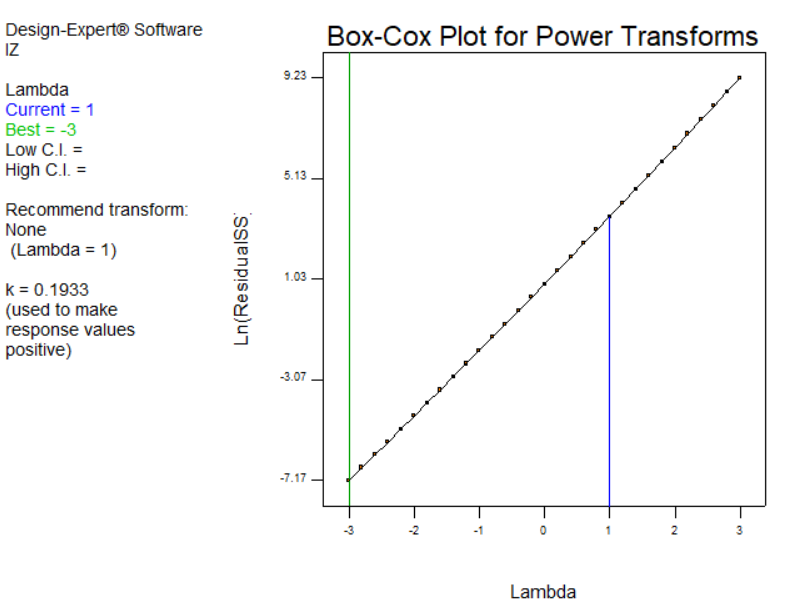


**c**


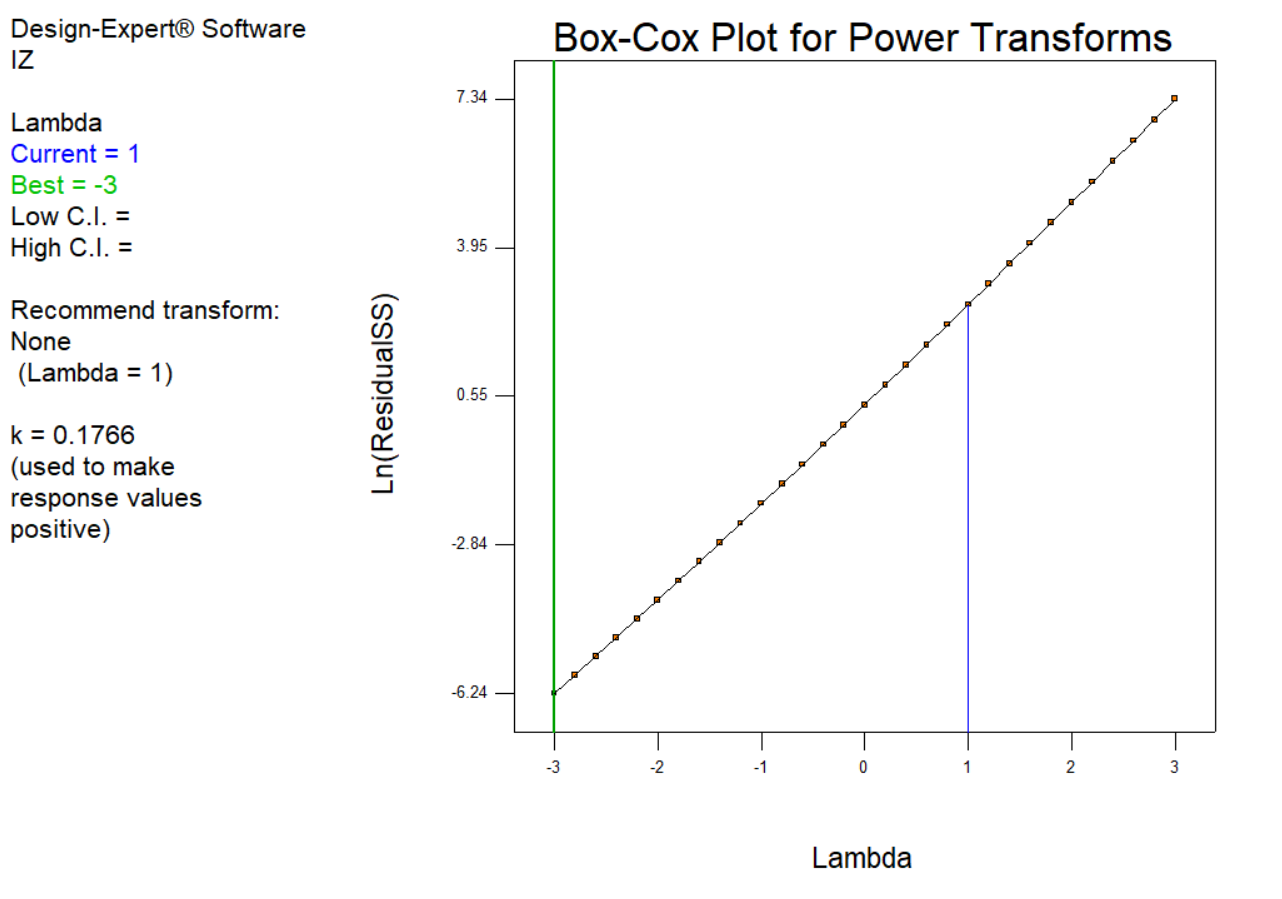
**Figure S2.** Model diagnostic plots **a** the normal probability plot of residuals for C6 metabolite **b** The normal probability plot of residuals for C11 metabolite **c** Box Cox plot for C6 metabolite, **d** Box Cox plot for C11 metabolite

**d**


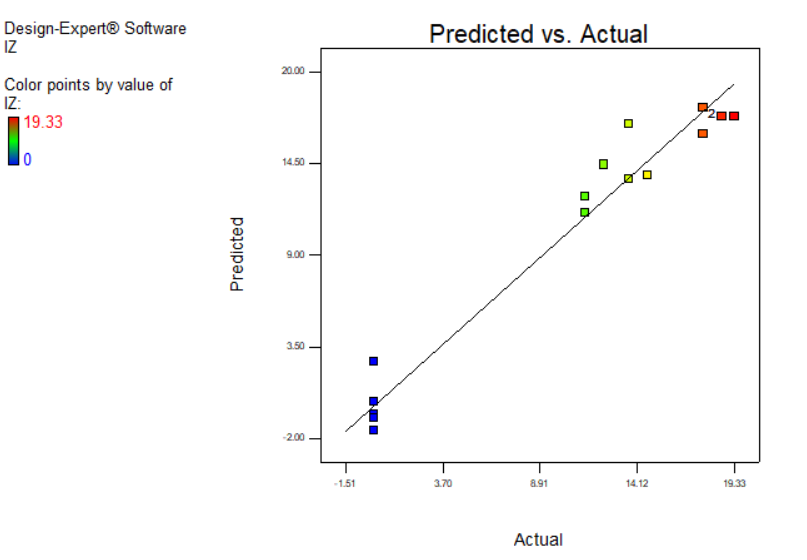


**a**


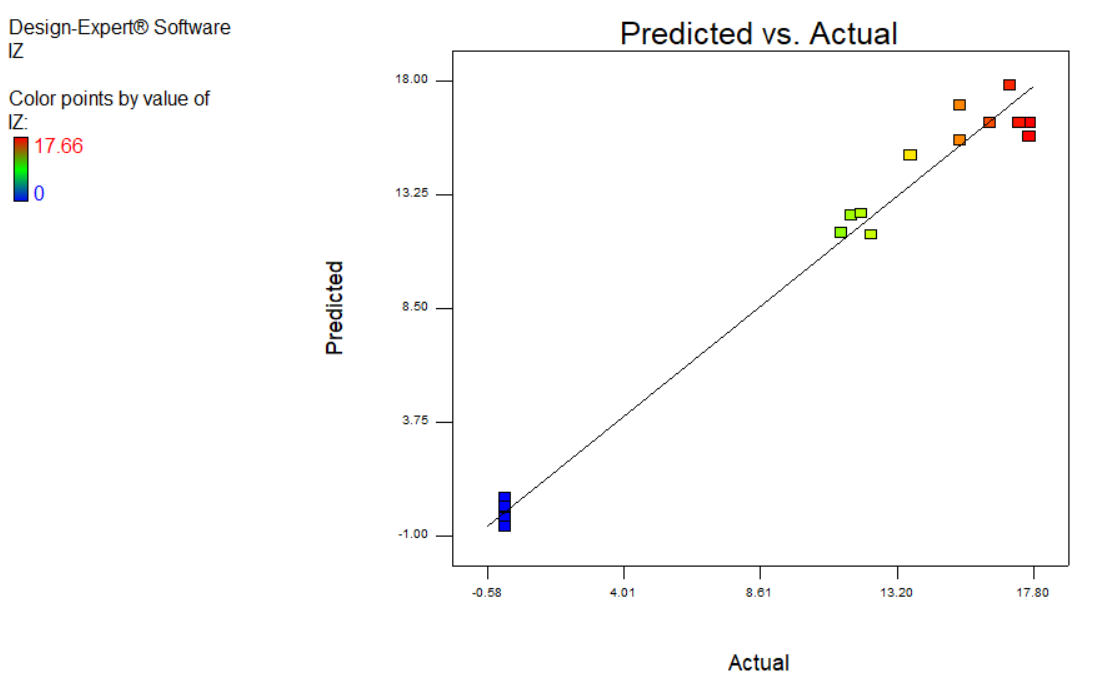


**b**


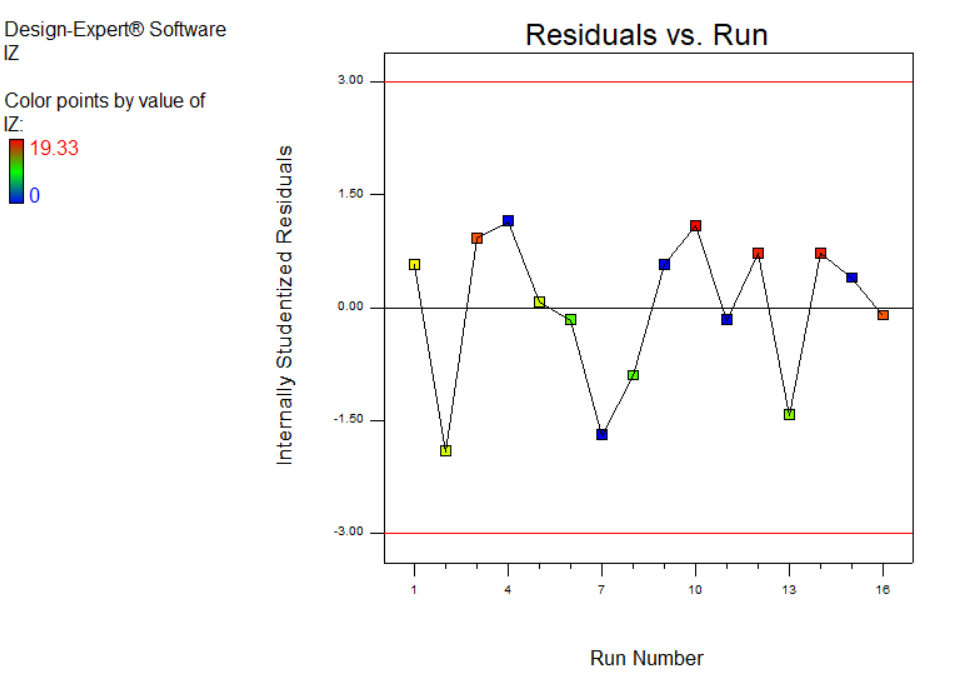


**c**


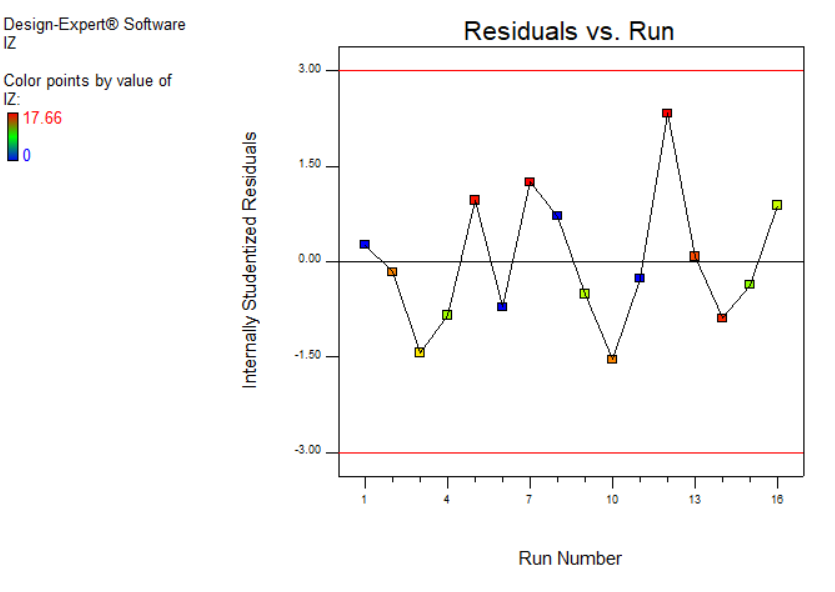


**d**

**Figure S3.** Model diagnostic plots **a** Predicted versus actual values plot for C6 metabolite and **b** Predicted versus actual values plot for C11 metabolite **c** Residuals versus Run number plot for C6 metabolite **d** Residuals versus Run number plot for C11 metabolite


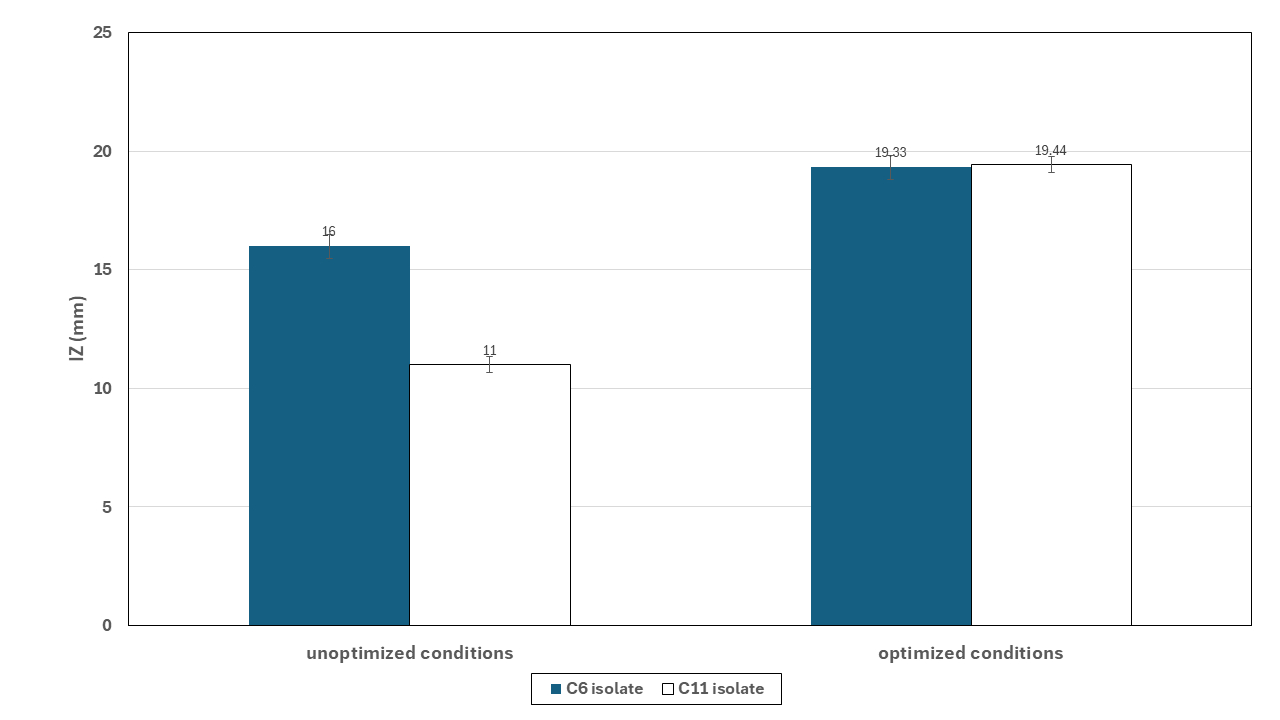


**Fig. S4.** Comparison of metabolite production by C6 isolate and C11 isolate using optimized and unoptimized conditions


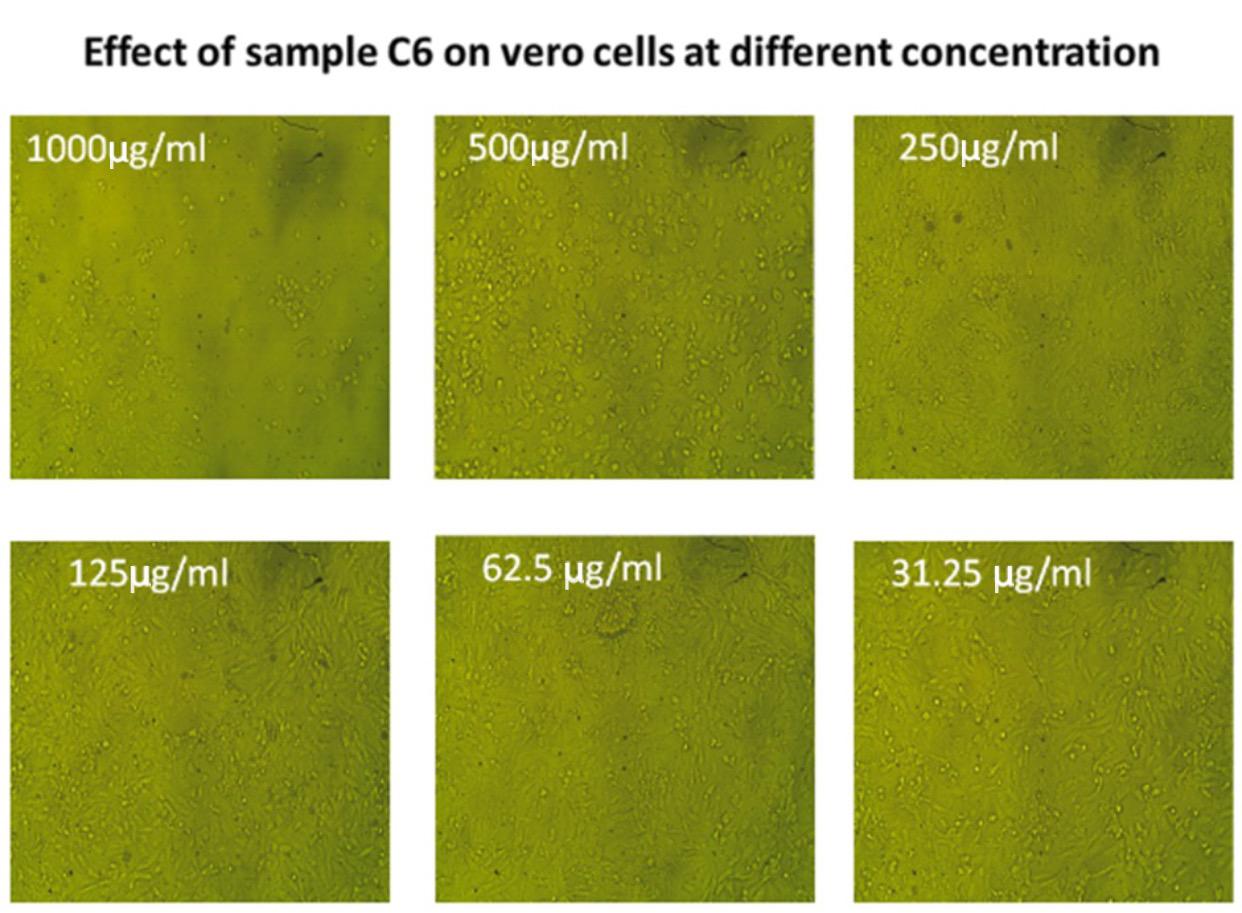


**(a)**


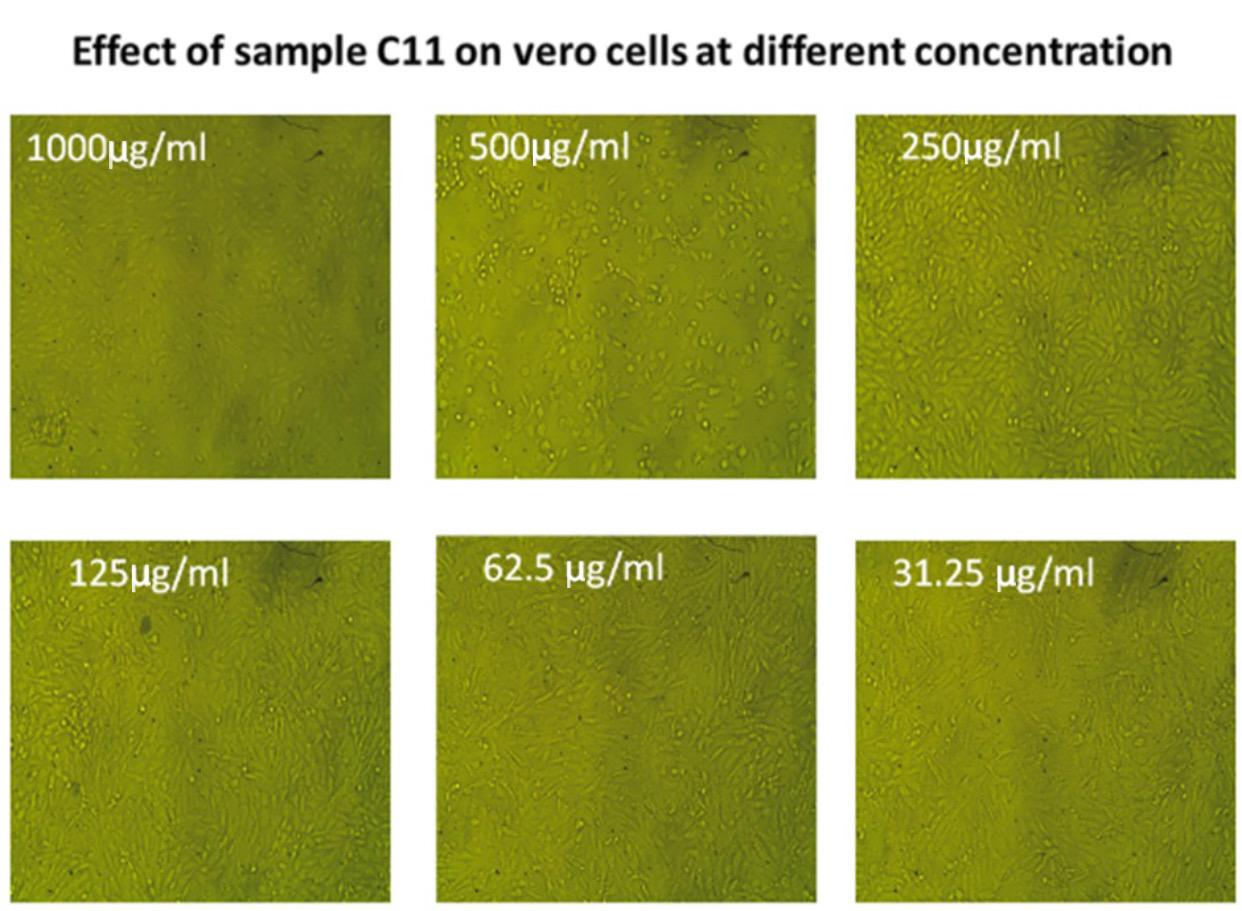


**(b)**


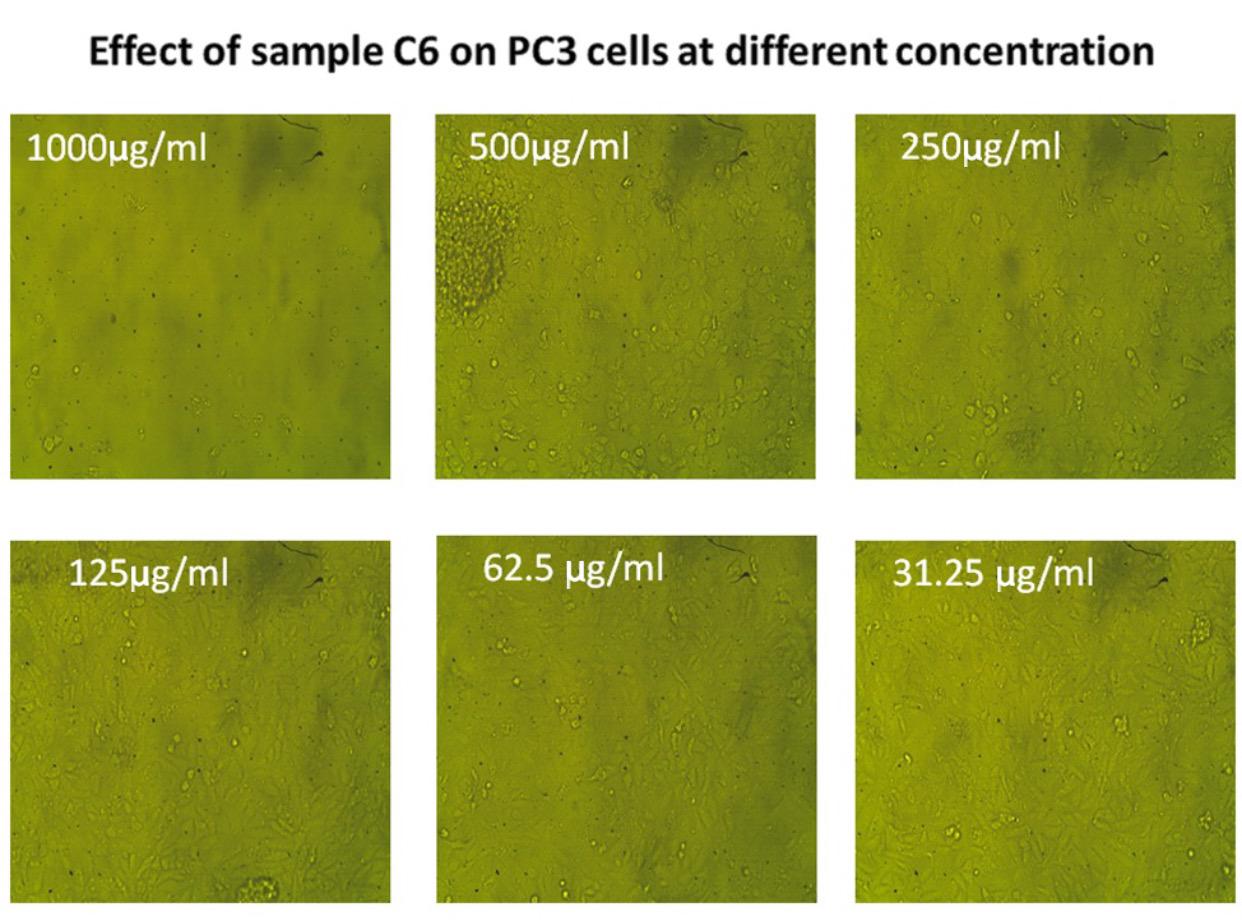


**(c)**


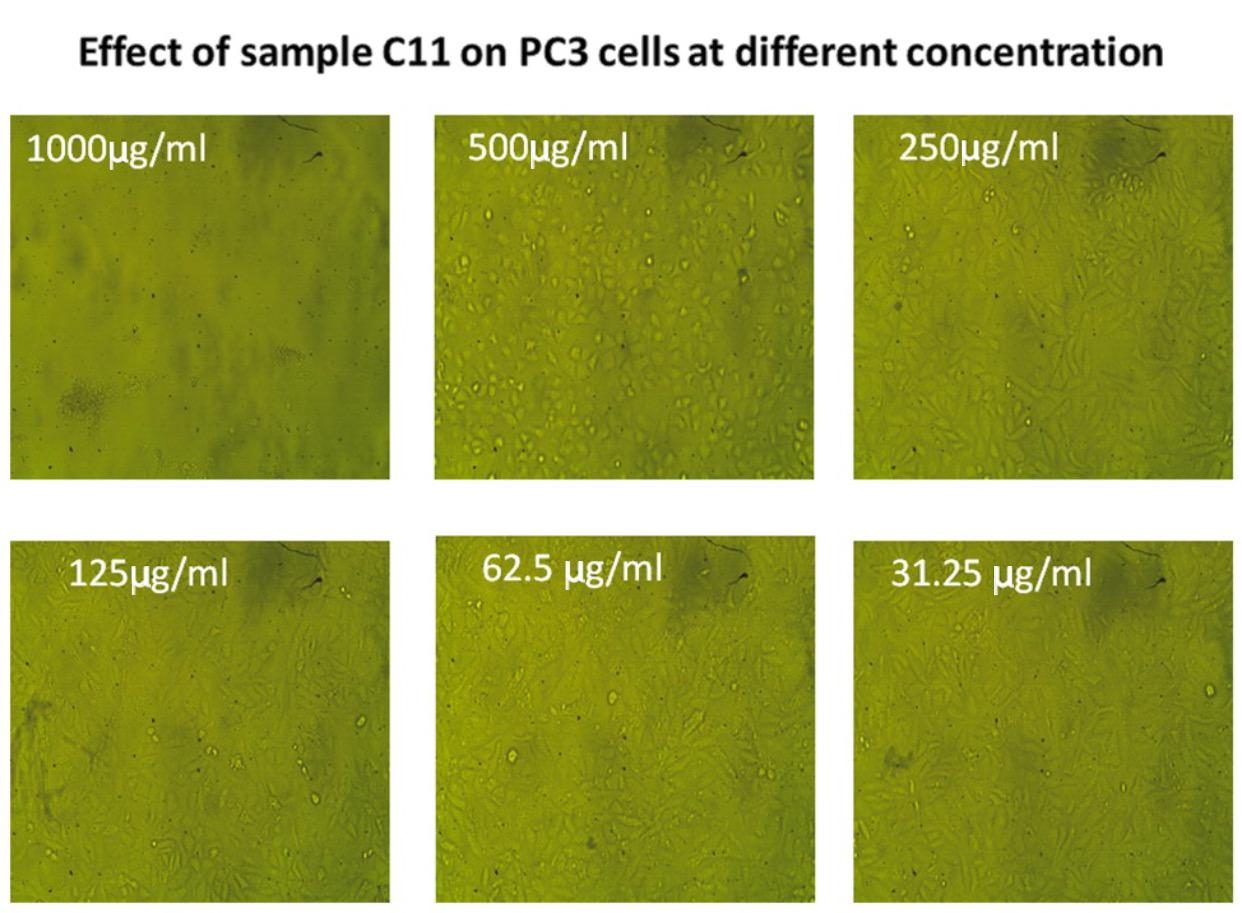


**(d)**

**Figure S5.** Effect of different concentrations (1000-31.25 µg/mL) of C6 and C11 extracts on Vero cell lines (a, and b, respectively); on PC3 cell lines (c, and d, respectively).
